# Characterizing Hydroxyl Radical Formation from the Light-Driven Fe(II)–Peracetic Acid Reaction, a Key Process for Aerosol-Cloud Chemistry

**DOI:** 10.1021/acs.est.3c10684

**Published:** 2024-04-15

**Authors:** Steven J. Campbell, Chris La, Qingyang Zhou, Jason Le, Jennyfer Galvez-Reyes, Catherine Banach, K. N. Houk, Jie Rou Chen, Suzanne E. Paulson

**Affiliations:** † Department of Atmospheric and Oceanic Sciences, University of California at Los Angeles, 520 Portola Plaza, Los Angeles, California 90095, United States; ‡ Department of Chemistry and Biochemistry, University of California, Los Angeles, California 90095, United States

**Keywords:** Fe(IV), iron photochemistry, pH dependence, kinetics model, Fenton chemistry, peroxy acids

## Abstract

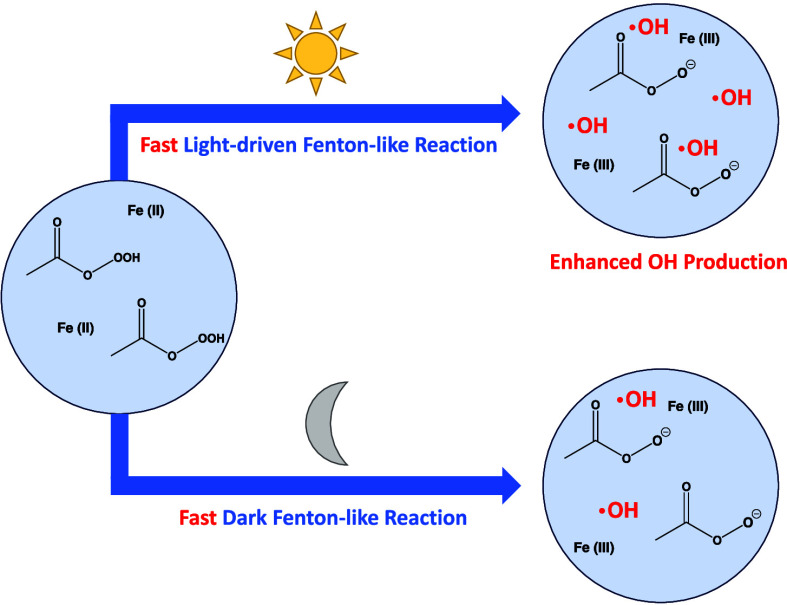

The reaction of peracetic acid (PAA) and Fe(II) has recently gained attention due to its utility in wastewater treatment and its role in cloud chemistry. Aerosol-cloud interactions, partly mediated by aqueous hydroxyl radical (OH) chemistry, represent one of the largest uncertainties in the climate system. Ambiguities remain regarding the sources of OH in the cloud droplets. Our research group recently proposed that the dark and light-driven reaction of Fe(II) with peracids may be a key contributor to OH formation, producing a large burst of OH when aerosol particles take up water as they grow to become cloud droplets, in which reactants are consumed within 2 min. In this work, we quantify the OH production from the reaction of Fe(II) and PAA across a range of physical and chemical conditions. We show a strong dependence of OH formation on ultraviolet (UV) wavelength, with maximum OH formation at λ = 304 ± 5 nm, and demonstrate that the OH burst phenomenon is unique to Fe(II) and peracids. Using kinetics modeling and density functional theory calculations, we suggest the reaction proceeds through the formation of an [Fe(II)–(PAA)_2_(H_2_O)_2_] complex, followed by the formation of a Fe(IV) complex, which can also be photoactivated to produce additional OH. Determining the characteristics of OH production from this reaction advances our knowledge of the sources of OH in cloudwater and provides a framework to optimize this reaction for OH output for wastewater treatment purposes.

## Abstract 2

We characterize the mechanism, pH, wavelength, and stoichiometric dependences of light-driven OH formation from the reaction of Fe(II) with organic peracids.

## 1 Introduction

In 1876, Fenton discovered a new oxidant system, later named the Fenton reaction. The eponymous Fenton and many other researchers spent their entire careers trying to understand the reaction mechanism.^1^ The subsequent debate over the identity of the oxidant lasted for decades; candidates included the hydroxyl radical (OH), the ferryl-oxo ion (Fe=O^2+^), and the perferryl-oxo ion (Fe=O^3+^). More than 100 years after its discovery, Sawyer and co-workers^2^ finally made a convincing case that the form of the oxidant under conditions relevant to the environment, where water and oxygen are ubiquitous, is the hydroxyl radical, OH. The mechanism, however, is most likely not the simple, oft-repeated form of the Fenton reaction
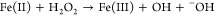
R1

Instead, it may well involve the metal ions being activated by the peroxide, which then reacts with O_2_ in the solution, producing superoxide (O_2_^•–^) or its protonated form (HO_2_). This, in turn, reacts with H_2_O_2_ to generate O(^1^D) (singlet oxygen), which abstracts a hydrogen atom from an available organic molecule, producing OH.^1^ However, there is also evidence that either Fe=O^2+^ or Fe=O^3+^ can abstract a hydrogen atom from water, OH can oxidize Fe(II), producing Fe(III), and H_2_O_2_ reduces Fe(III)to make Fe(II).^3^ The so-called “photo-Fenton” reaction has also been discussed; the mechanism involves the recycling of Fe(III) back to Fe(II) through the photoreduction of Fe(III) complexed with available organic ligands, typically driven by UV light.^4^

Large uncertainty remains regarding the chemistry and sources of OH in the cloud droplets. Models of the consumption of organics and other lines of evidence indicate that additional sources of OH are needed to explain observations.^5^ As part of a study to investigate OH radical formation in cloudwater, Paulson et al.^5^ found that when atmospheric aerosols are mixed with water and exposed to UV light, they produce an extremely rapid but short-lived burst of OH. The quantity of OH produced in the short burst, however, appeared to be larger than OH in cloud droplets from both the uptake of OH from the gas phase and the smaller bulk-chemistry sources (such as the Fenton reaction) under most conditions.^5^ Recently, additional research on chemistry taking place at the droplet interface indicates that this may be an additional strong source of OH radicals,^6−8^ comparable to the OH burst described in Paulson et al.,^5^ and to uptake from the gas phase.^7^ Aqueous OH is a key player in cloud droplet chemistry,^9,10^ contributing to the irreversible formation of secondary organic aerosol (SOA) upon cloud re-evaporation.^11,12^ Aerosol-cloud interactions and OH-mediated aqueous-phase processing of aerosol particles in cloud droplets can alter the size distribution, chemical composition, and radiative properties of aerosol particles (both by changing their size distribution and by forming brown carbon), therefore influencing their health-relevant properties, as well as their direct and indirect effects on climate.^10,11,13,14^ Further, these reactions may contribute substantially to the global production of sulfate, particularly by mediating reactions associated with dimethyl sulfide, notably the oxidation of methanesulfonic acid and dimethyl sulfoxide.^15−17^

Organic peroxides make up a large fraction of organic aerosols, up to 80%.^18^ Some of these peroxides are peracids; for example, Steimer et al.^19^ specifically identified the formation of monoperoxypinic acids from α-pinene ozonolysis. Peracids are a major product of OH-driven aldehyde oxidation under low NO_*x*_ conditions in the gas phase and oxidation reactions in the aqueous/condensed phases, either via a reaction between HO_2_ and RO_2_ radicals or via auto-oxidation and photolysis of compounds like biacetyl, and the chemistry associated with the peracid group is expected to be similar for PAA and larger peracids.^18^ Paulson et al.^5^ demonstrated that mixtures of peracetic acid (PAA) and Fe(II) produced similar behavior to that of the aerosol particles when mixed with acidified water. The reaction of PAA with Fe(II)
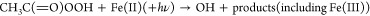
R2

At least partly due to the dangers of working with concentrated organic peroxides, the chemistry of peracids with iron has not been very widely studied, but as far as we are aware, no other examples of this dramatic chemistry have been observed.

R2 has recently been determined to have a rate constant of at least 1 × 10^5^ M^–1^ s^–1^ at pH 3,^20,21^ more than 3 orders of magnitude larger than the Fenton reaction (∼77 M^–1^ s^–1^).^22^ Furthermore, the OH yield from R2 was strongly photoenhanced, resulting in an OH yield of about 2, rather than ∼1 from the dark reaction of Fe(II) and PAA.^5^ Many characteristics of the Fe(II)-PAA reaction of relevance to the atmospheric community, including its pH and wavelength dependence and how the reaction depends on the stoichiometry of the reactants, are not known.

Dissolved iron concentrations in cloudwater are variable, typically ranging from 10^–7^ – 10^–4^ M,^23^ with Fe(II) constituting a substantial fraction of dissolved iron during both day and night.^24,25^ In the absence of H_2_O_2_ or organic peroxides, oxidation of aqueous Fe(II) to Fe(III) via the reaction with O_2_ is rate limiting and is relatively slow with *k* ∼ 1.4 × 10^–4^ M^–1^ s^–1^ at pH 4.^26^

In addition to arising from reactions within droplets and condensed phases,^18^ PAA is among the most abundant peroxides in the atmosphere, with gas phase concentrations observed as high as ∼1 ppb, second only to methyl hydroperoxide.^27,28^ PAA also has a sufficiently high Henry’s law coefficient, 837 M atm^–1^^29^, that appreciable concentrations in cloud water result from partitioning. However, recent work indicates that PAA may react at the surface, so the bulk chemistry investigated here may be of limited importance. The rapid reaction of PAA with Fe(II) (dark *k* = 0.1–1 × 10^5^ M^–1^ s^–1^ from pH 7 to pH 3)^20,21^ may represent a previously unrecognized source of significant OH production.

In parallel to developing interest in R2 in the atmospheric chemistry community, the wastewater community has recently recognized the potential for the PAA reaction with Fe(II) to be a more powerful oxidizing approach than Fenton chemistry, itself popular because it is both effective and results in residues that cause less contamination issues than other oxidants, such as those containing halogens.^14^ The Fe(II) PAA reaction has emerged as a powerful oxidant that is superior to the Fenton reaction R1, as the rate constant of Fe(II) + PAA is 5 × 10^4^ M^–1^ s^–1^^21^ at circumneutral pH, compared to that of Fe(II) + H_2_O_2_ (77 M^–1^ s^–1^).^22^R2 likely activates faster due to (1) the lower Gibbs free energy of formation (Δ*G*_f_) associated with Fe(II) + PAA (−299.8) compared to Fe(II) + H_2_O_2_ (−118.5),^21^ (2) reduced bond energy of O–OH for PAA (88.4 kcal mol^–1^) compared to H_2_O_2_ (90.4 kcal mol^–1^),^21,30^ and (3) higher reduction potential of PAA (1.96 V) compared to H_2_O_2_ (1.76 V).^31−33^ Given the recent emergence of the Fe/PAA advanced oxidation system, uncertainty remains regarding the key reactive intermediates that are responsible for contaminant degradation. In addition, studies have probed the Fe(II)-PAA reaction system under different conditions, for instance, with and without UV irradiation,^21,34,35^ at different pHs,^21^ photolyzing PAA in the absence of transition metals,^36^ as well as using different iron/ligand/PAA combinations to activate PAA.^37,38^ Therefore, developing our understanding of the Fe(II)-PAA reaction, in particular its ability to produce OH, will aid in tailoring the conditions required to optimize this reaction for wastewater applications.

In this work, we explore several aspects of the reaction of PAA with Fe(II) R2. We tested several other metals and organic peroxides for similar chemistry. We characterize the pH and wavelength dependence of the Fe(II) reaction with PAA. We use a kinetic model and density functional theory calculations to probe the mechanism of the Fe(II) PAA reaction and develop insights into the reaction mechanism.

## 2 Materials and Methods

### 2.1 Reagents

All reagents were purchased from Sigma-Aldrich unless otherwise stated. Disodium terephthalate (>99%) and 2-hydroxyterephthalate (>98%) for OH quantification and calibration, respectively. Fe(II)SO_4_ (≥99%), Fe(III)SO_4_ (>99%), Mn(II)SO_4_ (∼99%), Cu(II)SO_4_ (99.999%), Cu(I)Cl (99.995%), Pb(II)SO_4_ (99.995%), and Sn(II)Cl (≥99.99%) were used for PAA-metal experiments. All solutions were prepared in Milli-Q water (>18 MΩ·cm). pH values were adjusted by adding appropriate volumes of 0.1N H_2_SO_4_ (Fisher).

### 2.2 Quantification of OH

OH was quantified using the terephthalate probe (TA).^39^ Excess aqueous TA (10 mM) reacts with OH to produce the highly fluorescent product 2-hydroxyterephthalate (hTA), which is then detected at λ_ex_/λ_em_ = 320/420 nm by using a fluorometer (Lumina, Thermo Scientific). Fluorescence measurements were acquired with a time resolution of 500 ms. For experiments investigating OH formation from the light-driven reaction of Fe(II) and PAA, known concentrations of Fe(II) and PAA were added stepwise to a 10 mM solution of TA mixed in a falcon tube for 5 s to ensure mixing but limit reaction before analysis. Then, 200 μL was immediately transferred to the fluorometer and illuminated with 320 nm light for 270 s.

Measurements of dark OH were performed using a high-performance liquid chromatography (LC) column coupled to a fluorescence detector (Shimadzu RF-10AXL detector), where reactions reach completion and are separated prior to fluorescence detection of hTA. Known concentrations of Fe(II) PAA reaction mixtures (200 μL) were transferred to the LC at different time intervals from a dark vial to get time-resolved dark OH formation. We consistently observe somewhat different yields from the different devices to measure fluorescence, likely due to slightly different wavelengths of the light sources and detectors. Most experiments were carried out at pH 3.5, but the effect of pH values up to 7 was also explored. hTA yields are variable as a function of pH, and OH concentrations were calculated using pH-dependent hTA yields as discussed in Gonzalez et al.^39^

### 2.3 Chemical Kinetics Model

The kinetic model developed in this study describing the chemistry of aqueous Fe(II) and PAA is presented in Table S1. It includes 85 individual reactions describing the reactions between Fe(II) and PAA (dark chemistry), as well as inorganic aqueous Fe(II)/Fe(III)/Fe(IV) chemistry. It also includes aqueous reactive oxygen species (ROS—OH, HO_2_, H_2_O_2_, O_2_^•–^) reactions, terephthalate probe chemistry for measuring OH, and photolysis reactions of Fe(OH)_2_^+^, H_2_O_2_, and PAA. Reactions and rate constants were synthesized from the literature and are referenced appropriately in Table S1. The reaction set builds upon earlier models and testing of those models against experimental data by our group and others.^22,39−41^ The kinetics model is solved using the Kinetics Pre-Processor (KPP) version 2.2.3,^42^ utilizing the Rosenbrock solver and gFortran compiler.

### 2.4 Density Functional Theory (DFT) Calculations

All calculations were carried out using the Gaussian 16 program.^43^ According to previous benchmarking works, the geometries were optimized using the PBE0^44,45^ functional with the def2-SVP^46^ basis set with the IEEPCM solvent model^47^ to describe the water environment. Grimme’s dispersion correction with damping^48,49^ was also used. All of the complex structures studied here are confirmed to be high-spin species (i.e., sextet for Fe(III), quintet for Fe(II)). Single point energies were calculated using the PBE0 functional with the D3(BJ) dispersion correction, def2-TZVPP basis set^46^, and SMD solvent model.^50^ The absorption spectrum is calculated using TD-DFT^51^ at the same level as a single point, and 20 states were calculated. Quasiharmonic^52^ and concentration corrections to enthalpy and entropy were made using Paton’s GoodVibes software.^53^ For the hydronium ion formed in the reaction, proton solvation energy reported by Kelly et al. was used,^54^ while the thermodynamic correction of a free proton in the gas phase was calculated using the Fermi–Dirac distribution.^55^

## 3 Results and Discussion

### 3.1 Physical and Chemical Drivers of the OH Burst

#### 3.1.1 Exploring OH Bursts from a Range of Transition Metals and Organic Peroxides

**Figure 1:**
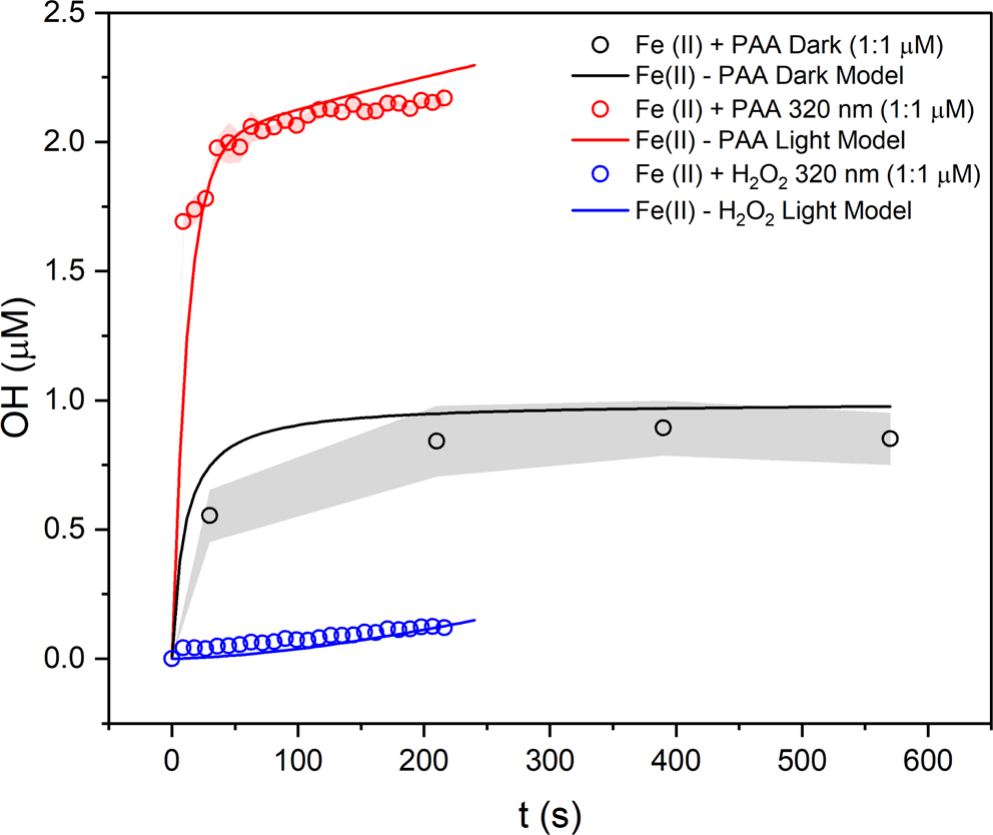
Comparison between the kinetic model (solid lines) and experimental results (circles) of the Fe(II) PAA reaction at pH 3.5 illuminated with 320 nm light. Also shown is the reaction of Fe(II) with H_2_O_2_ at pH 3.5. Shaded areas represent the standard deviation observed over 3 experimental repeats. Modeling results are discussed in Section 3.1.6.

Paulson et al.,^5^ demonstrated that the Fe(II)-PAA reaction when exposed to UV light produces an OH burst, a behavior similar to ambient particle samples in the same study. Here, we expand on this and explore a matrix of atmospherically relevant transition metals and peroxides to determine whether an OH burst is produced. Figure 1 shows the 1:1 μM reaction of Fe(II), H_2_O_2_, and PAA exposed to 320 nm of light. The OH burst is characterized as the rapid formation of OH, which ceases abruptly, typically within a few minutes, presumably because reactants are consumed.^5^ This behavior is observed for the Fe(II)-PAA reaction, for which a 1:1 μM Fe(II)-PAA mixture exposed to 320 nm UV light produces 1.98 ± 0.13 μM OH. This behavior is not observed for the Fe(II) + H_2_O_2_ reaction (Figure 1). We also explored the OH burst associated with the reaction of Fe(II) and 3-chloroperbenzoic acid, a commercially available peracid containing the same α-carbonyl hydroperoxyl group but with a different carbon backbone compared to PAA. Interestingly, a 1:1 μM mixture of Fe(II) and 3-chloroperbenzoic acid produces 2.11 ± 0.23 μM OH, matching the yield for PAA of OH within error (Figure S1). This result illustrates that larger peracids also produce the OH burst, which implies that organic peracids present in aerosol particles likely exhibit the same behavior and contribute to the OH burst phenomenon.

Reaction mixtures of Fe(II) with a range of commercially available peroxides with different functionalities, including cumene hydroperoxide, benzoyl peroxide, and *t*-butyl hydroperoxide, do not produce the OH burst. This suggests that the presence of the α-carbonyl in the peracid moiety is essential to produce the rapid light-driven OH burst. In addition, a range of redox-active transition metals that have been observed in ambient aerosol particles were tested for their ability to produce an OH burst when mixed with PAA. We probed the ability of different transition metals to produce the light-driven OH burst using a 1:1 reaction mixture of PAA with Fe(II), Fe(III), Cu(I), Cu(II), Pb(II), Mn(II), and Zn(II) (Figure S2). The OH burst is only observed for the Fe(II)-PAA reaction. Little to no OH formation was observed for the mixtures of other metals with PAA. Therefore, these results highlight the specific importance of the reaction of Fe(II) with PAA and peracids regarding the OH burst mechanism, and we focus on this reaction forthwith.

#### 3.1.2 Exposure to UV Light Enhances the OH Burst

An equimolar reaction mixture of Fe(II) PAA at 1:1 μM in the dark results in the formation of 0.89 ± 0.11 μM OH. However, when the reaction is exposed to 320 ± 5 nm UV light at a near-atmospheric photon flux of 2 × 10^15^ cm^–2^ nm^–1^ s^–1^, OH formation increased by a factor of more than 2, with 1.98 ± 0.13 μM OH produced. This is consistent with the data presented in Paulson et al.^5^ and clearly demonstrates that exposure of this reaction system to UV light dramatically enhances OH formation.

**Figure 2:**
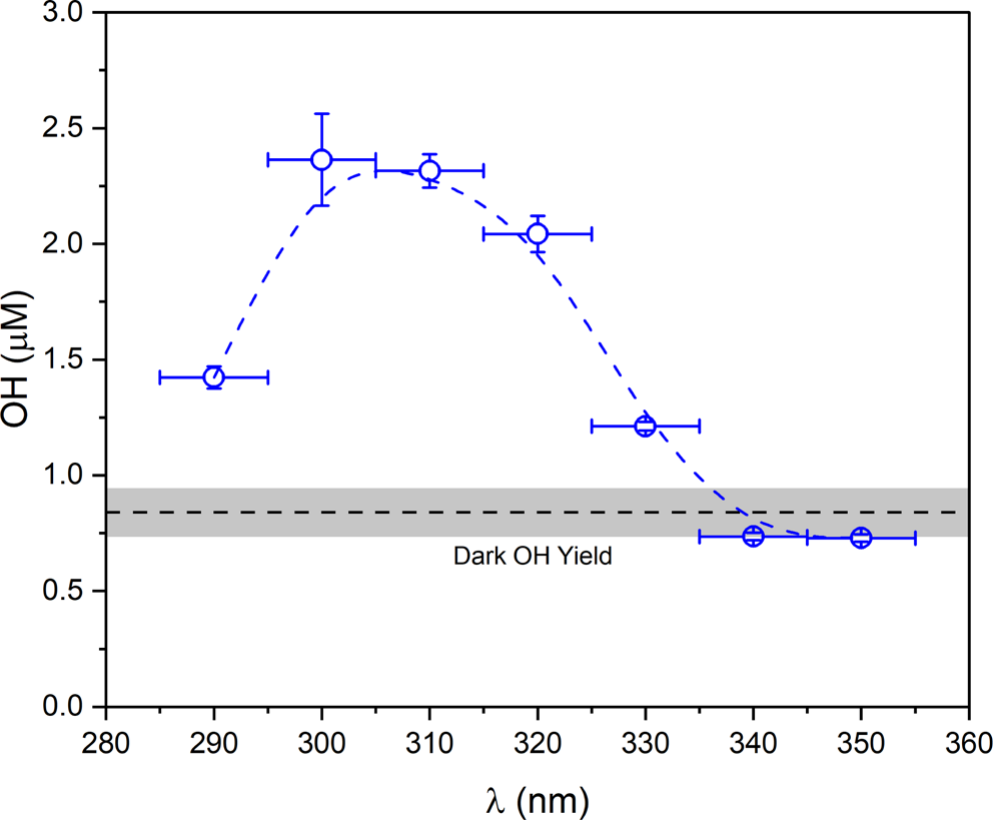
Action spectrum showing OH production from 1:1 μM Fe(II) PAA as a function of exposure wavelength, where the reaction mixture is exposed to light for 147 s. *Y*-error bars represent the standard deviation observed over three experimental repeats, and *X*-error represents the 10 nm slit width during light exposure in the fluorometer. The horizontal black dashed line shows the measured dark OH yield from the Fe(II) PAA reaction, and the gray shaded area represents the standard deviation observed over three experimental repeats.

To investigate the influence of UV light on this mechanism further, 1:1 μM reaction mixtures of Fe(II) and PAA were exposed to different wavelengths of light. It should be noted that hTA calibrations were performed at each wavelength to account for different hTA fluorescence efficiencies at different excitation wavelengths. We observe a strong dependence on exposure light wavelength; OH yields for 1:1 μM PAA reactions exposed to λ = 290–350 nm (considering the lower limit of UV radiation at the Earth’s surface ∼295 nm) are displayed in Figure 2.

The OH yield from this reaction reaches a maximum when at λ = 304 ± 5 nm, producing 2.36 ± 0.19 μM OH, and decreases as a function of both increasing and decreasing UV wavelength around the observed λ_max_. The additional OH yield of the light-driven reaction decreases to about 0 at λ = 340 nm and above, where OH production is equal to the dark yield of OH. Additionally, the OH yield decreases as a function of increasing wavelength, with an observed OH yield of 1.42 ± 0.04 μM at λ= 290 nm. The OH yield is roughly equivalent to dark OH formation, within error, at λ > 340 ± 5 nm. This demonstrates the strong dependence of the light-driven burst of OH radicals on the wavelength of light and that this reaction will be efficiently photoenhanced at tropospherically relevant wavelengths of light that cloud droplets are exposed to.

#### 3.1.3 Concentration and pH Dependence of the Fe(II)-PAA Reaction

**Figure 3:**
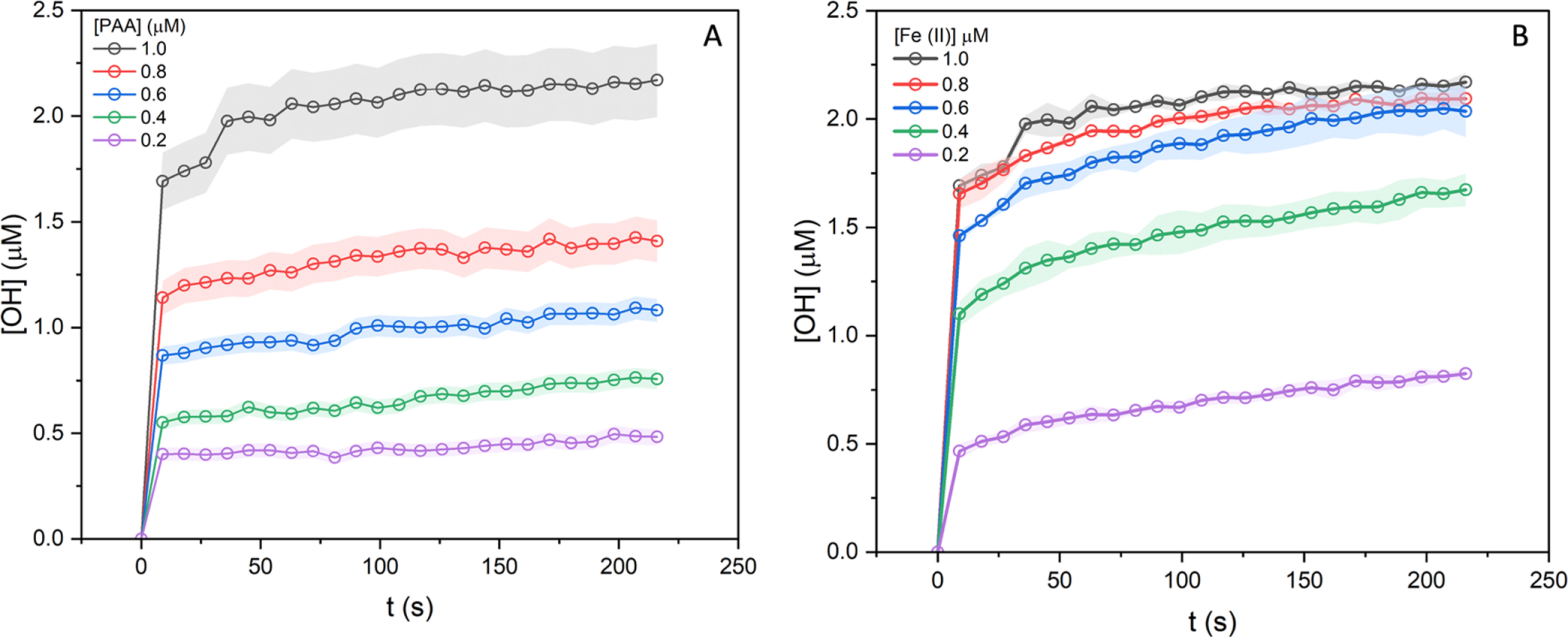
OH bursts from the Fe(II)-PAA reaction as a function of concentration for (A) PAA (with Fe(II) held constant at 1 μM) and (B) Fe(II) (with PAA held constant at 1 μM). Shaded areas represent the standard deviation over three experimental repeats.

**Figure 4:**
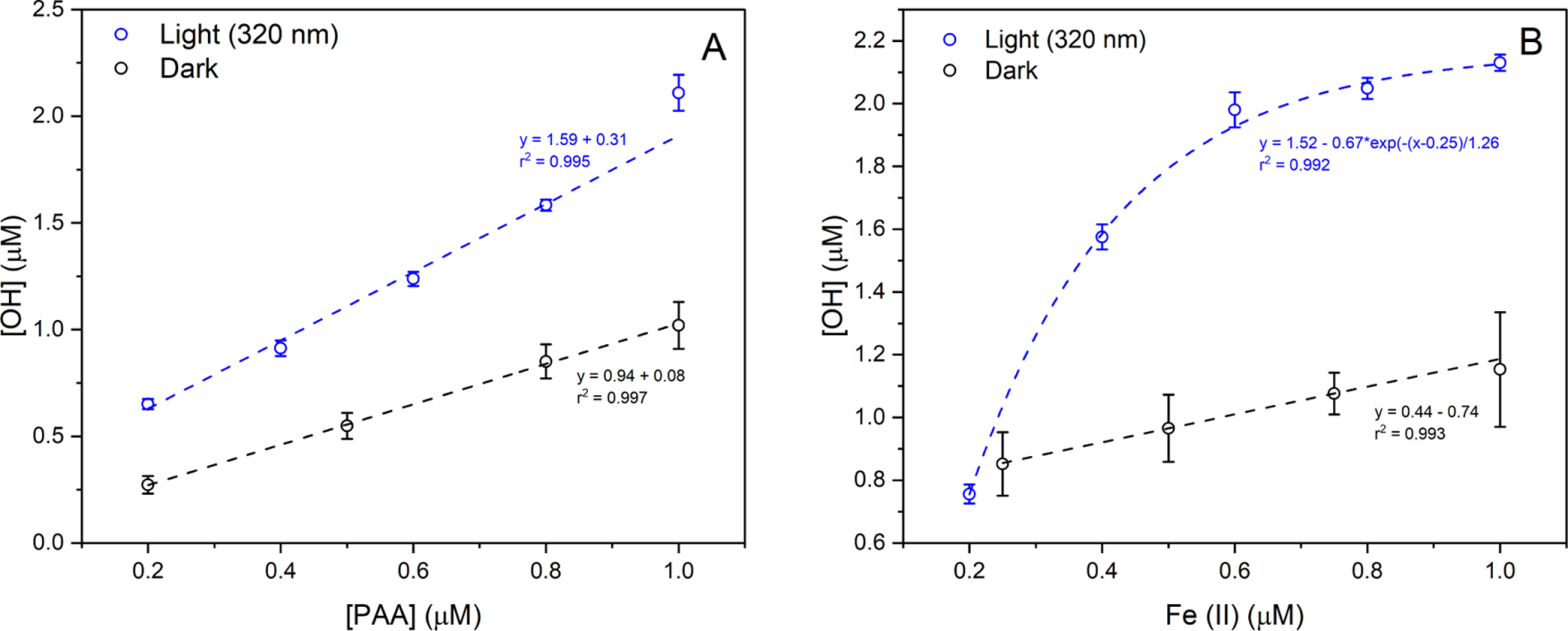
Concentration dependence of light-driven (λ = 320 ± 5 nm) and dark OH yields observed after 21 s as a function of (A) PAA concentration with Fe(II) kept constant at 1 μM and (B) Fe(II) concentration with PAA kept constant at 1 μM. Error bars represent the standard deviation as observed over three experimental repeats.

The stoichiometry/concentration dependences of both the light-driven and dark Fe(II)-PAA reactions are presented in Figures 3 and 4. Concentration dependence of both Fe(II) and PAA were probed between 0 and 1 μM. This concentration range was selected as dissolved iron concentrations in cloudwater are variable but have been detected in concentrations ranging from 10^–7^ – 10^–4^ M,^23^ with Fe(II) constituting a substantial fraction of dissolved iron in the daytime.^24^

We were unable to find reports of measurements of PAA in cloudwater. PAA has Henry’s law constant of (837 M atm^–1^),^29^ and measured gas phase concentrations as high as 1 ppb,^27,28^ so in the absence of significant sinks, its concentrations in cloudwater could feasibly be as high as several hundred nM. Given its extremely rapid reaction with Fe(II), however, it seems likely that it should be consumed as soon as it is absorbed. Highly viscous aerosol particles can potentially stabilize reactive species,^56,57^ and thus, the concentration of PAA or other organic peracids present in an aerosol may be higher immediately after the particle dissolves upon activation to become a cloud droplet. While not yet investigated, it is also possible that the “burst” chemistry takes place in particles when they deliquesce.

Figures 3 and 4 show both the light-driven (320 nm) and dark OH burst when varying PAA concentrations from 0 to 1 μM in the presence of 1 μM Fe(II) (Figures 3A and 4A) and varying Fe(II) concentrations from 0 to 1 μM in the presence of 1 μM PAA (Figures 3B and 4B). It is apparent that in addition to the burst, there is some additional OH formation that lasts for a minimum of several minutes and that exceeds the formation of OH from the Fenton reaction R1. This secondary phase varies for different stoichiometries and appears to be more dependent on the PAA concentration than on the Fe(II) concentration (Figure 3). This is consistent with the additional formation of OH from organic radicals that are formed in the initial reaction, which is discussed below in the kinetics modeling section. Comparison of the concentration dependence of the light-driven Fe(II)-PAA reaction, as well as the dark Fe(II)-PAA reaction, are shown in Figure 4A,B, respectively. This plot shows data from the initial burst after 21 s and does not include slower phase chemistry observed after this time point.

The dependence on the stoichiometry of OH formation from R2 in both the dark and light (λ = 320 nm) experiments exhibit different behaviors (Figure 4). The light-driven reaction is approximately linear if equivalent concentrations of Fe(II) and PAA are maintained (Figure S3). Increasing PAA while holding Fe(II) constant does not increase OH production; if anything, there is a slight reduction in yield as the PAA/Fe(II) stoichiometry increases (Figure S4). The dark Fe(II)-PAA reaction is approximately linear with respect to both Fe(II) and PAA concentration. The light Fe(II)-PAA reaction is also approximately linear with respect to PAA concentration but nonlinear with respect to Fe(II) concentration. The nonlinear dependence for Fe(II) in the light implies that the iron is catalytic, potentially indicating a photoreduction step in the mechanism. The dark data, however, also suggests a catalytic mechanism because while OH production increases linearly, 0.25 μM Fe(II) is sufficient to produce an OH concentration of ∼0.8 μM, and increasing the iron 4-fold only increases the OH concentration to 1.85 μM. This seems to imply that a complex with one iron and around three molecules of PAA may be responsible for the light-driven chemistry involved.

The dependence on the PAA concentration in the dark is stoichiometric; within error, the OH produced equals the initial concentration of the PAA after about 200 s. In the presence of light, however, OH production is slightly larger than twice the initial concentration of PAA, and the OH/PAA ratio increases somewhat as the PAA concentration increases. This implies a mechanism that includes multiple PAA molecules complexing each iron, something that would become more likely as the ratio of PAA/Fe increases and more ligands on one iron are more likely to produce OH. This can be rationalized by the absorption cross-section of Fe substantially increasing as the metal center ligates with more PAA molecules; this is consistent with ligand-to-metal charge transfer (LMCT) upon Fe-PAA complex formation, which increases the absorption efficiency of the complex relative to the individual metal and ligand. This is also consistent with the notion that there is a multiligand process involved in the light reactions: the UV absorption spectra in Paulson et al.^5^ show that the iron complex is somewhat less than stoichiometric; the absorption spectrum indicates there was about 3.5 μM Fe(III) from a 5:5 μM PAA/Fe(II) reaction mixture.

#### 3.1.4 pH Dependence of the Fe(II)-PAA Reaction

**Figure 5:**
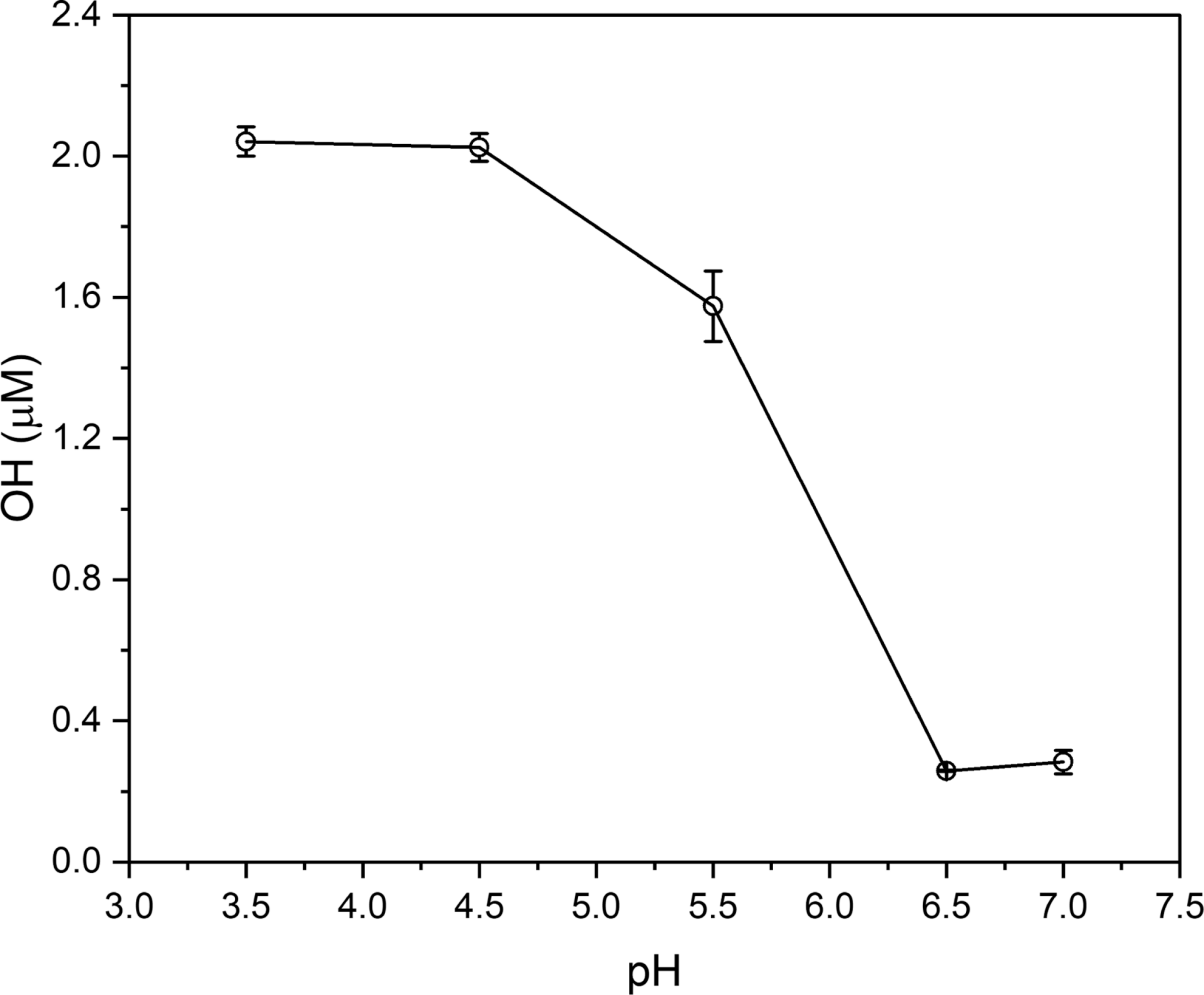
OH yield of equimolar 1:1 Fe(II)-PAA reaction over different pHs typically observed in ambient cloudwater. Error bars represent the standard deviation observed over three experimental repeats.

Figure 5 shows the pH dependence of OH production from the reaction of 1:1 μM PAA with Fe(II) over the range pH 3.5–7. The OH formation yield is constant from pH 3.5 to 4.5 at slightly above 2 μM but drops dramatically as the pH approaches 6.5 to about 0.3 μM. PAA has a p*K*_a_ of 8.2 at 25 °C,^58^ so PAA should remain predominantly in its neutral state across this pH range. Modeling of Fe(II) and Fe(III) speciation using Minteq software (Figures S5 and S6) shows that Fe(II) speciation remains largely unchanged over the pH range 3–7; however, soluble Fe(III) decreases as a function of increasing pH, existing almost entirely in its insoluble precipitate form Fe(OH)_2_^+^, and therefore will not participate in aqueous redox chemistry. Kim et al.^21^ measured the rate constant for the Fe(II)-PAA reaction, showing it decreases by about 1 order of magnitude from pH 3 to 8.1 (from *k* = 1 × 10^5^ M^–1^ s^–1^ at pH 3 to *k* = 0.1 × 10^5^ M^–1^ s^–1^ at pH 8.1).^20,21^ Lower OH formation yields are observed at pH > 6 when illuminated with light. The observed yield is also lower than that observed for the dark OH reaction at pH 3.5 (Figure 1), which provides evidence that the dark Fe(II)-PAA reaction is suppressed at higher pH.

#### 3.1.5 Kinetics Modeling of the Light-Driven Fe(II) PAA Reaction

The kinetic model describing the dark reaction between Fe(II)-PAA, as well as a range of aqueous inorganic reactions and photochemistry, is presented in Table S1. Comparison between model and experimental OH formation from the 1:1 μM Fe(II)-PAA reaction in both the dark and light (pH 3.5, λ_ex_ = 320 nm) is presented in Figure 1 (dashed lines). The model results are in good agreement with both the dark and light-driven chemistry of Fe(II) and PAA.

Regarding the dark reaction, the experimental data is well described using rate constants for the initial Fe(II)-PAA reaction, which at pH 3.5 is *k* ∼ 1.05 × 10^5^ M^–1^ s^–1^.^21^ The model captures the plateau observed from *t* = 40 s in the experimental data, as well as being in good agreement with the experimental yield of 0.89 ± 0.11 μM. The initial reaction of the Fe(II) can proceed through the following proposed routes:
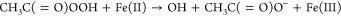
R3
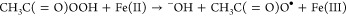
R4
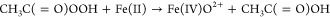
R5where either OH is formed with the corresponding acetate anion (CH_3_C(=O)O^–^) (R3), or ^–^OH is formed with the corresponding acetylperoxy radical (CH_3_C(=O)O^•^) (R4). “Fe(IV) could also be formed, but large uncertainty remains regarding the chemistry of Fe(IV)O^2+^; EPR spectroscopy is usually used to detect Fe(IV), but it cannot differentiate OH from Fe(IV), so it cannot be used to probe Fe(IV)from this reaction.^59−61^

Our dark kinetics model for OH formation has a best fit when ∼40% of the reaction proceeds through R3, while ∼60% proceeds through either R4 or R5. To the authors’ knowledge, this is the first estimate of the branching ratio of the Fe(II)-PAA reaction. As indicated by our DFT calculations, the formation of the acetylperoxy radical CH_3_C(=O)O^•^ via R4 is likely favored over OH via R3 by 16.4 kcal/mol, which may explain why this branching ratio slightly in favor of R4 best fits the dark experimental data in Figure 1, also in agreement with previous studies.^21,37,62^ In addition to OH directly produced through R3, the model also considers a range of other possible routes to OH formation through radical chemistry involving CH_3_C(=O)O^•^ formed through R4. In brief, upon formation, CH_3_C(=O)O^•^ promptly undergoes decarboxylation to form the methyl radical (^•^CH_3_) (RS14, Table S1, *k* = 2.5 × 10^5^ s^–1^), which in turn rapidly reacts with O_2_ to form the methyl peroxy radical (CH_3_OO^•^) (*k* = 2.8–4.1 × 10^9^ M^–1^ s^–1^).^63^ CH_3_OO^•^ can then undergo a range of recombination and radical reactions that, in competition with other pathways, can lead to the production of OH, as well as H_2_O_2_ and HO_2_ that may participate in further reactions that also contribute to OH production (RS16–RS27, Table S1).^64−66^ These radical species (OH, ^•^CH_3_, ^•^CH_3_COO, ^•^CH_3_C(=O)O) were all identified in a recent study using EPR spectroscopy to examine radicals produced from the Fe(II)-PAA reaction.^14^

In addition to interpreting dark Fe(II)-PAA chemistry, the kinetic model was applied to determine the photochemical mechanism that is responsible for the factor ∼2 enhancement of OH production. We considered two possible mechanisms for the light-driven enhancement of the OH burst in this relatively simple chemical system which only involves Fe(II), PAA, and products from this R1 the formation of a Fe(III) acetate complex, which can potentially undergo photoreduction via photo-Fenton-like chemistry, and (2) direct formation of an [Fe(II)(H_2_O)_2_(PAA)_2_] complex that subsequently photoactivates to produce OH, as discussed later in Section 3.1.6.

Kinetics modeling results for pathway 1 (Figure S7) and several other lines of evidence indicate this pathway does not explain the observed OH burst. This mechanism considers the following steps, in line with classical photo-Fenton-like chemistry
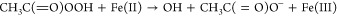
R6
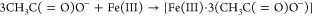
R7
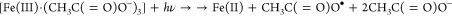
R8wherein in R6, Fe(III) and CH_3_C(=O)O^–^ are produced alongside the OH radical. In principle, Fe(III) and CH_3_C(=O)O^–^ can form an iron acetate complex. Model runs, including Fe(III)-acetate binding under these conditions (pH 3.5) using MINTEQ (Figure S6), show the limited formation of iron acetate complexes at pH 3.5. This is likely due to the p*K*_a_ of acetic acid being 4.76, so only ∼5% exists in its dissociated acetate form at pH 3.5. In addition, monocarboxylates such as the acetate ligand are, in general, much poorer at forming complexes than bidentate carboxylates, such as oxalate; in the presence of acetate, the formation of Fe(OH)^2+^ dominates.^67^ The absorption cross-section of Fe(III)-Acetate was reported to be σ = 9.96 × 10^–18^,^68^ which assuming a quantum yield of Φ = 1 and the measured photon flux of *F* = 2 × 10^15^ cm^–2^ nm^–1^ s^–1^,^5^ leads to a photolysis frequency of *J* = 1.99 × 10^–2^ s^–1^. Enhanced photochemistry of Fe(III)-carboxylate complexes is generally more pronounced than the solvated Fe(III) and carboxylates due to the possibility of metal-to-ligand charge transfer (LMCT) excitations in the former, which typically have an increased absorption cross-section and thus are more likely to undergo photochemistry.^69^ Despite the enhanced *J* associated with the formation of Fe(III)-acetate, the limited formation of Fe(III)-acetate included in the model, therefore, does not describe the observed OH burst. In addition, experiments performed where Fe(III) and acetate ligands were mixed at pH 3.5 in the presence of TA and illuminated with 320 nm light did not produce an observable OH burst. Therefore, this mechanism likely does not describe the photoenhanced OH production in the Fe(II)-PAA reaction. Direct photolysis of other constituents in the reaction mixture such as Fe(OH)^2+^, PAA, and H_2_O_2_ (RS80–82, Table S1), at concentrations present in this series of experiments, also does not occur fast enough to explain the factor of ∼2 increase in light-driven OH formation.

However, a comparison of a model run with pathway (2) in Figure 1 shows a good fit between the model and experimental OH production from the Fe(II) PAA reaction. This mechanism considers the formation of [Fe(II)(H_2_O)_2_(PAA)_2_] (see Section 3.1.6), assuming the complexation of bidentate PAA ligands is diffusion-limited. The model was optimized to determine the photolysis efficiency of this reaction, with *J* = 8 × 10^–2^ s^–1^ and associated σ = 4 × 10^–17^ cm^2^, assuming Φ = 1 and known *F* = 2 × 10^15^ cm^–2^ nm^–1^ s^–1^.^5^ While this absorption cross-section is relatively high, it is on the same order as that observed for Fe(II)-oxalate complexes.^70^ In addition, the complexation of H_2_O_2_ with Fe(II) has been proposed as a potential mechanism of the Fenton reaction.

#### 3.1.6 DFT Calculations of Fe-PAA Complexes

**Figure 6:**
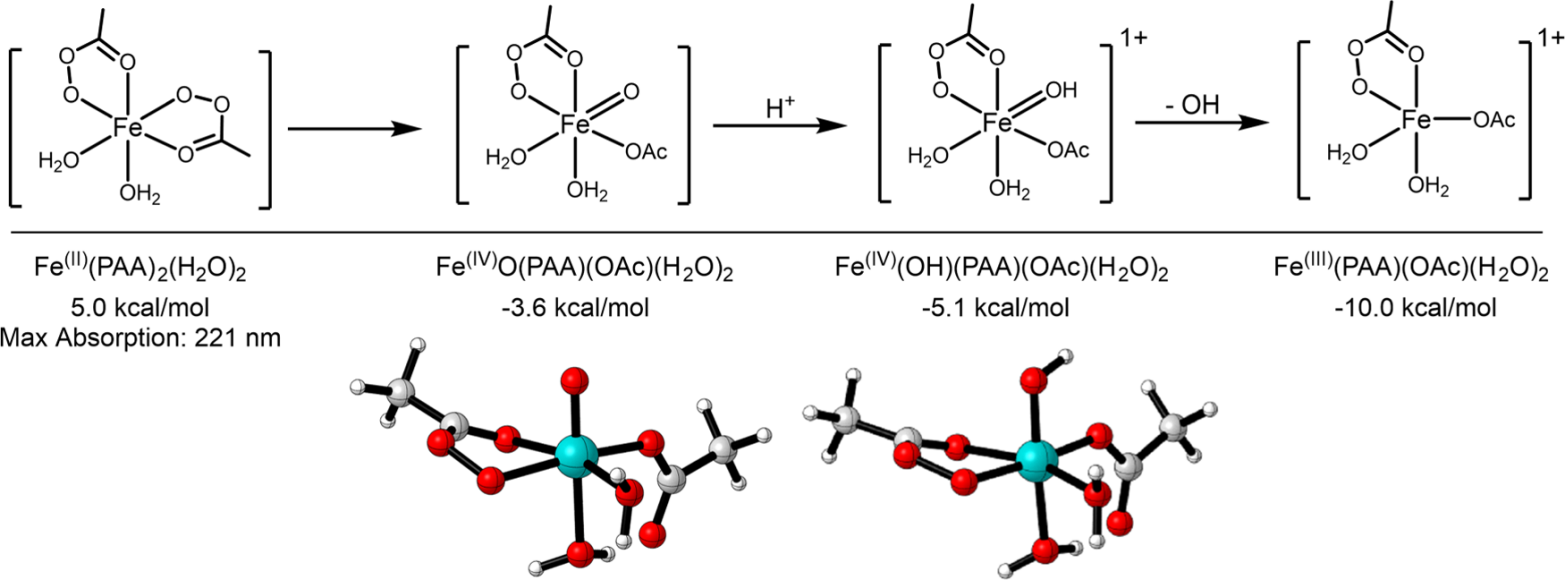
Relative free energies of potential Fe(II)(PAA)_2_ complexes. The free energies and UV–vis absorption maxima were calculated using PBE0-D3(BJ)/def2-TZVPP/SMD(water)//PBE0-D3(BJ)/def2-SVP/IEEPCM(water) under the standard state (298 K, 1 M).

To determine whether the formation of the Fe(II)-PAA complex in aqueous media is feasible, DFT calculations were performed. The relative free energies of formation (Δ*G*_f_) of potential Fe(III) complexes are displayed in Figure S8. Fe(III) is predicted to readily form a stable complex with PAA in aqueous media, where the relative Δ*G*_f_ is −25.6 kcal mol^–1^ for Fe(III)(PAA)_3_ compared to Fe(OH_2_)_6_. This also results in a substantial red shift of the absorption wavelength of the Fe complex from λ_max_ = 275 nm for Fe(II)(OH_2_)_6_ to λ_max_ = 369 nm for Fe(III)(PAA)_3_. Our experimental results show that there is no light-driven enhancement of OH formation above 340 nm. However, the formation of an [Fe(II)(PAA)_2_(H_2_O)_2_] complex has a relatively low Δ*G*_f_ of 5 kcal mol^–1^ (Figure 6). One peroxide bond of a PAA ligand then breaks and forms a Fe(IV) complex, [Fe(IV)O(PAA)(OAc)(H_2_O)_2_], which has a predicted λ_max_ = 315 nm (see Figure S9), in reasonable agreement with the experimental action spectra (λ_max_ = 304 ± 5 nm) (Figure 2). Therefore, we hypothesize that this species is photoactivated to produce additional OH. Note that our calculations were conducted at standard state (1 M of all reactants, including H^+^), i.e., pH = 0. As pH increases and the concentration of protons decreases, the protonation of Fe(IV)O(PAA)(OAc)(H_2_O)_2_ is suppressed (Figure 6). Δ*G =* −1.5 kcal mol^–1^ at pH 0, and increases to Δ*G =* 3.3 kcal mol^–1^ and Δ*G =* 7.3 kcal mol^–1^ at pH 3.5 and 6.5, respectively. This reduced protonation also inhibits the subsequent release of OH via this proposed mechanism (Figure 6), which agrees with our experimental observations, which show decreasing OH formation at increasing pH (Figure 5).

## 4 Atmospheric and Environmental Implications

Aerosol-cloud interactions represent one of the largest uncertainties with respect to our understanding of the climate system. OH-mediated chemistry in the aqueous phase is a key driver of cloudwater chemistry, promoting the formation of SOA with different physical and chemical properties upon cloud re-evaporation. However, models of OH formation in cloudwater have indicated there has been a missing source (or sources) of OH.^5,9^

This work heavily suggests that the light-driven OH burst observed when aerosol particles take up water is a unique phenomenon between peracids and Fe(II).^4^ The OH burst is not observed for a range of atmospherically relevant transition metals or for a range of hydroperoxides and organic peroxides. We do, however, observe the OH burst when Fe(II) is mixed with PAA, as well as 3-chloroperbenzoic acid, the only other commercially available peracid. This strongly suggests that species containing peracid groups in SOA contribute to OH burst chemistry. Some PAA may be among those peracids, as it may form in particles or be incorporated into them during homogeneous nucleation events.

The strong photoenhancement in the presence of UV light between 300–330 nm would also suggest that the OH burst has more influence during daytime cloud chemistry. The overall OH yield is expected to be lower, ∼1.5, however, because the availability of photons from sunlight increases rapidly from 290–300 nm, and the higher energy photons produce less OH. However, because a substantial OH burst is also observed in the absence of light, the “dark burst” of OH formation is also likely important.

Organic peracids have been detected in biogenic and anthropogenic SOA;^19^ they are multigeneration oxidation products in ambient SOA, formed when volatile organic compounds are oxidized by OH or O_3_. While they are relatively reactive, reactive species have been shown to be preserved in viscous SOA particles.^56^ This could preserve substantial concentrations of peracids, which upon interaction with a cloud droplet, liberate peracids upon dissolution, which in the presence of Fe(II) (which has a typical concentration of 10^–7^ – 10^–4^ M in ambient cloudwater) represents an additional large source of OH in cloudwater.^23^ Fang et al. recently observed enhanced OH formation (although not an OH burst) from Fenton-like reactions of isoprene hydroxy hydroperoxide from isoprene SOA, demonstrating that SOA components can engage in Fenton chemistry at faster rates than Fe(II) + H_2_O_2_.^71^

DFT calculations performed suggest that organic peracids can effectively coordinate with iron and facilitate the reaction by forming a more stable carboxylic acid product. Moreover, the coordination also leads to a substantial red shift of the absorption properties of the Fe complex, moving the absorption into the atmospherically relevant wavelength range, potentially leading to the light-driven OH burst. Regarding this reaction’s application in wastewater treatment, we show that illumination with UV light increases OH production substantially by a factor of 2 and that more acidic pH favors OH formation, which likely enhances the efficacy of this reaction for removing organic contaminants. We highlight that OH is the dominant radical formed from this reaction under these experimental conditions through a thermodynamically favored pathway R2, which is informative when determining how this reaction decomposes organic contaminants in wastewater and understanding the chemistry that leads to byproducts from these reactions in wastewater.

Finally, the Fenton Reaction R1 has been the subject of intense study for over a century, and its chemistry is still not completely understood. For this reason, we have focused on trying to characterize the behavior of the reaction under different conditions so that its atmospheric implications can be understood and modeled and so that it can be most effectively used as an oxidant to destroy toxic compounds in wastewater treatment.

## Supporting Information Available

Detailed reactions used in kinetic modeling; range of experiments showing the OH burst; Minteq modeling results; additional kinetic modeling results; and additional DFT results (PDF)
