# Supplementary material for: Characterizing Hydroxyl Radical Formation from the Light-Driven Fe(II)–Peracetic Acid Reaction, a Key Process for Aerosol-Cloud Chemistry
Source: Environ Sci Technol. 2024 Apr 15;58(17):7505–15. doi: 10.1021/acs.est.3c10684 (PMC11064221; doi:10.1021/acs.est.3c10684)
Supplement: Supplementary file 1 — es3c10684_si_001.pdf [file es3c10684_si_001.pdf]

Supplementary Information For:

Characterizing Hydroxyl Radical Formation from the Light-driven Fe(II) - Peracetic Acid Reaction, a Key Process for Aerosol-Cloud Chemistry

Steven J. Campbell,[§†] Chris La, [§¥] Qingyang Zhou,[‡] Jason Le,[§] Jennyfer Galvez-Reyes,[§] Catherine Banach,[§] K. N. Houk,[‡] Jie Rou Chen[§ʃ] and Suzanne E. Paulson [§*]

[§]Department of Atmospheric and Oceanic Sciences, University of California at Los Angeles, 520 Portola Plaza, Los Angeles, California, 90095, United States

[‡] Department of Chemistry and Biochemistry, University of California, Los Angeles, California, 90095, United States

[†] Now at: MRC Centre for Environment and Health, Environmental Research Group, Imperial College London, 86 Wood Lane, London W12 0BZ, UK.

[¥] Now at: Department of Chemistry, University of California Berkeley, Berkeley, CA 94720

[ʃ]Now at: South Coast Air Quality Management District, Diamond Bar, CA 91765

* Corresponding author: paulson@atmos.ucla.edu

This file contains: 21 pages, 1 Table, 9 Figures.

Table S1 – Reactions included in the Fe (II) PAA model.

Class	No.	Reaction	k_f (M^{-1} s^{-1}, s^{-1})	k_b	K_{eq}	ref
**PAA**	S1	$CH_3C(O)OOH + Fe\ (II) \rightarrow CH_3COO^- + \bullet OH + Fe\ (III)$	0.4×10^5			[1] + This work
**PAA**	S2	$CH3C(O)OOH + Fe\ (II) \rightarrow CH_3C(O)O\bullet + {}^-OH + Fe\ (III)$	1.1×10^5			[1] + This Work
**PAA**	S3	$CH_3C(O)OOH + Fe\ (II) \rightarrow CH_3C(O)OH + Fe(IV)O^{2+}$	---			[1]
**PAA**	S4	$CH_3C(O)OOH + Fe\ (III) \rightarrow CH_3C(O)OO\bullet + Fe\ (II) + H^+$	2.72			[1]
**PAA**	S5	$CH_3(O)OOH + CH_3C(O)O\bullet \rightarrow CH_3C(O)OO\bullet + CH_3C(O)OH$	----			[1] [2]
**PAA**	S6	$CH_3C(O)OOH + \bullet OH \rightarrow CH_3C(O)OO\bullet + H_2O$	9.33×10^8			[2]
**PAA**	S7	$CH3C(O)OOH + \bullet OH \rightarrow CH_3(O)C\bullet + H_2O + O_2$	9.33×10^8			[2]
**PAA**	S8	$CH3C(O)OOH + \bullet OH \rightarrow CH_3C(O)OH + HO_2\bullet$	9.33×10^8			[2]
**PAA**	S9	$CH_3C(O)OOH + 2\ Fe(IV)O_2^+ \rightarrow CH_3C(O)O^- + 2\ Fe\ (III) + {}^-OH + O_2$	----			[3]
**PAA**	S10	$CH_3(O)C\bullet + O_2 \rightarrow CH_3C(O)OO\bullet$	2.5×10^9			[3]
**PAA**	S11	$CH_3C(O)OO\bullet + CH_3C(O)OO\bullet \rightarrow 2\ CH_3C(O)O\bullet + {}^3O_2$	8.3×10^9			[3]
**PAA**	S12	$CH_3C(O)OO\bullet + HO_2\bullet \rightarrow CH_3C(O)OH + {}^3O + {}^3O_2$	2×10^6			[3]
**PAA**	S13	$CH_3C(O)OO\bullet \rightarrow HO_2\bullet + CH_2CO$	1.82 s^{-1}			[3]
**PAA**	S14	$CH_3C(O)O\bullet \rightarrow \bullet CH_3 + CO_2$	2.3×10^5 s^{-1}			[2]
**PAA**	S15	$\bullet CH_3 + O_2 \rightarrow CH_3OO\bullet$	$(2.8\text{-}4.1)\times10^9$			[2]
**PAA**	S16	$CH_3OO\bullet + CH_3OO\bullet \rightarrow HCHO + CH_3OH + O_2$	2.8×10^8			[4]
**PAA**	S17	$CH_3OO\bullet + CH_3OO\bullet \rightarrow 2HCHO + H_2O_2$	3.4×10^8			[3]
**PAA**	S19	$CH_3OO\bullet + HO_2\bullet \rightarrow CH_3OH + O + O_2$	5×10^8			[3]
**PAA**	S20	$CH_3OO\bullet + CH_3OO\bullet \rightarrow 2CH_3O\bullet + O_2$	1.8×10^8			[4]
**PAA**	S21	$\bullet OCH_3 \rightarrow \bullet CH_2OH$	5×10^5 s^{-1}			[4]
**PAA**	S22	$\bullet CH_2OH + O_2 \rightarrow \bullet OOCH_2OH$	4.9×10^9			[4]
**PAA**	S23	$\bullet OOCH_2OH + \bullet \bullet OOCH_2OH \rightarrow \bullet OCH_2OH + \bullet OCH_2OH + O_2$	8.9×10^8			[3]
**PAA**	S24	$\bullet OOCH_2OH + \bullet OOCH_2OH \rightarrow 2HCOOH$	1.6×10^9			[3]
**PAA**	S25	$\bullet OOCH_2OH + HO_2\bullet \rightarrow CH_2(OH)_2 + O + O_2$	2.0×10^6			[3]
**PAA**	S26	$\bullet OOCH_2OH \rightarrow HO_2\bullet + HCHO$	10 s^{-1}			[5]
**PAA**	S27	$\bullet OCH_2OH \rightarrow HCHO + HO\bullet$ (b-scission)	1×10^6 s^{-1}			[3]
**H_2O_2**	S28	$H_2O_2 + Fe\ (II) \rightarrow {}^-OH + \bullet OH$	63-76			[6]
**H_2O_2**	S29	$H_2O_2 + Fe\ (II) \rightarrow Fe(IV)O^{2+} + H_2O$	63-76			[7]
**H_2O_2**	S30	$H_2O_2 + Fe\ (III) \rightarrow HO_2\bullet + Fe\ (II) + H^+$	0.01-0.001			[8,9]
**H_2O_2**	S31	$H_2O_2 + \bullet OH \rightarrow HO_2\bullet + H_2O$	3.3×10^7			[6,10,11]
**H_2O_2**	S32	$H_2O_2 + 2\ Fe(IV)O_2^+ \rightarrow HO_2\bullet + Fe^{3+} + {}^-OH$	1×10^4			[12]
**ROS**	S33	$OH^\cdot + OH^\cdot \rightarrow H_2O_2$	5.5×10^9			[13]
**ROS**	S34	$H_2O_2 + OH^\cdot \rightarrow HO_2^\cdot + H_2O$	3.2×10^7			
**ROS**	S35	$O_2^{\cdot -} + OH^\cdot \rightarrow OH^- + O_2$	1.01×10^{10}			[13]
**ROS**	S36	$HO_2^\cdot + OH^\cdot \rightarrow H_2O + O_2$	7.1×10^9			[13]
**ROS**	S37	$O_2^{\cdot -} + H_2O_2 \rightarrow OH^- + OH^\cdot + O_2$	0.13			[13]
**ROS**	S38	$O_2^{\cdot -} + O_2^{\cdot -} \rightarrow O_2 + H_2O_2 - 2H^+$	6.0×10^5			
**ROS**	S39	$HO_2^\cdot + O_2^{\cdot -} \rightarrow HO_2^- + O_2$	9.7×10^7			
**ROS**	S40	$H_2O_2 + HO_2^\cdot \rightarrow H_2O + O_2 + OH^\cdot$	0.5			

**ROS**	S41	$HO_2^{\cdot} + HO_2^{\cdot} \rightarrow O_2 + H_2O_2$	8.3×10^5			13
**ROS**	S42	$O_2^{2-} + H^+ \rightarrow HO_2^-$	1×10^{10}			13
**ROS**	S43	$HSO_4^- + OH^{\cdot} \rightarrow SO_4^{\cdot -} + H_2O$	3.5×10^5			
**EQUILIBRIA**	S44	$H_2O \rightleftharpoons H^+ + OH^-$	1.3E-3	1.3E11	1E-14	
**EQUILIBRIA**	S45	$H_2O_2 \rightleftharpoons H^+ + HO_2^-$	1.26E-2	1E10	1.26E-12	
**EQUILIBRIA**	S46	$HO_2 \rightleftharpoons H^+ + O_2^{\cdot -}$	1.14E6	7.2E10	1.58E-5	
**EQUILIBRIA**	S47	$H^+ + SO_4^{2-} \rightleftharpoons HSO_4^-$			9.77E1	
**Fe(II)/Fe (III)**	S48	$Fe^{3+} + H_2O \rightleftharpoons FeOH^{2+} + H^+$			6.11E-3	14
**Fe(II)/Fe (III)**	S49	$FeOH^{2+} + H_2O \rightleftharpoons Fe(OH)_2^+ + H^+$			7.78E-6	14
**Fe(II)/Fe (III)**	S50	$Fe^{2+} + H_2O \rightleftharpoons FeOH^+ + H^+$			3.16E-10	14
**Fe(II)/Fe (III)**	S51	$Fe^{3+} + SO_4^{2-} \rightleftharpoons FeSO_4^+$			8.32E3	14
**Fe(II)/Fe (III)**	S52	$Fe^{3+} + 2SO_4^{2-} \rightleftharpoons Fe(SO_4)_2^-$			2.63E5	14
**Fe(II)/Fe (III)**	S53	$Fe^{2+} + SO_4^{2-} \rightleftharpoons FeSO_4$			1.78E2	14
**Fe(II)/Fe (III)**	S54	$Cl^- + Fe^{3+} \rightleftharpoons FeCl^{2+}$	3E3	2.16E3	1.39	14
**Fe(II)/Fe (III)**	S55	$Fe^{2+} + O_2 = Fe^{3+} + O_2^{\cdot -}$	1E-4 (1<pH<4, 37°C) n			14
**Fe(II)/Fe (III)**	S56	$Fe(III)^* + O_2^{\cdot -} = Fe^{2+} + O_2$	5E7			14
**Fe(II)/Fe (III)**	S57	$FeSO_4^+ + O_2^{\cdot -} = Fe^{2+} + SO_4^{2-} + O_2$	<1E3^			14
**Fe(II)/Fe (III)**	S58	$Fe(SO_4)_2^- + O_2^{\cdot -} = Fe^{2+} + 2\,SO_4^{2-} + O_2$	<1E3^			14
**Fe(II)/Fe (III)**	S59	$Fe(III)^* + HO_2^{\cdot} = Fe^{2+} + O_2 + H^+$	2E4			14
**Fe(II)/Fe (III)**	S60	$FeSO_4^+ + HO_2^{\cdot} = Fe^{2+} + SO_4^{2-} + O_2 + H^+$	<1E3^			14
**Fe(II)/Fe (III)**	S61	$Fe(SO_4)_2^- + HO_2^{\cdot} = Fe^{2+} + 2SO_4^{2-} + O_2 + H^+$	<1E3^			14
**Fe(II)/Fe (III)**	S62	$Fe^{3+} + H_2O_2 \rightleftharpoons Fe(HO_2)^{2+} + H^+$	3.1E7	1E10	3.1E-3	14
**Fe(II)/Fe (III)**	S63	$FeOH^{2+} + H_2O_2 \rightleftharpoons Fe(OH)(HO_2)^+ + H^+$	2E6	1E10	2E-4	14
**Fe(II)/Fe (III)**	S64	$Fe(II)^* + OH^{\cdot} = Fe^{3+} + OH^-$	2.7E8			14
**Fe(II)/Fe (III)**	S65	$FeSO_4 + OH^{\cdot} = Fe^{3+} + SO_4^{2-} + OH^-$	2.7E8			14
**Fe(II)/Fe (III)**	S66	$Fe(II)^* + O_2^{\cdot -} = Fe^{3+} + O_2^{2-}$	1E7			14
**Fe(II)/Fe (III)**	S67	$FeSO_4 + O_2^{\cdot -} = Fe^{3+} + SO_4^{2-} + O_2^{2-}$	5E8			14
**Fe(II)/Fe (III)**	S68	$Fe(II)^* + HO_2^{\cdot} = Fe^{3+} + HO_2^-$	1.2E6			14
**Fe(II)/Fe (III)**	S69	$FeSO_4 + HO_2^{\cdot} = Fe^{3+} + SO_4^{2-} + HO_2^-$	1.2E6			14
**Fe(II)/Fe (III)**	S70	$Fe^{2+} + H_2O_2 = Fe^{3+} + OH^{\cdot} + OH^-$	55			14
**Fe(II)/Fe (III)**	S71	$FeOH^+ + H_2O_2 = Fe^{3+} + OH^{\cdot} + 2OH^-$	55			14
**Fe(II)/Fe (III)**	S72	$FeSO_4 + H_2O_2 = Fe^{3+} + SO_4^{2-} + OH^{\cdot} + OH^-$	78			14
**Fe(II)/Fe (III)**	S73	$Fe(HO_2)^{2+} = HO_2^{\cdot} + Fe^{2+}$	2.3E-3			14
**Fe(II)/Fe (III)**	S74	$Fe(OH)(HO_2)^+ = Fe^{2+} + HO_2^{\cdot} + OH^-$	2.3E-3			14
**Fe(II)/Fe(III)**	S75	Fe (III) + $CH_3C(O)O$- ⇌ Fe (III)-OOCCH3	2.8E3	0.14	19E4	15
**Fe (IV)**	S76	$Fe(IV)O^{2+} + H_2O \rightarrow HO\bullet + HO- + Fe(III)$	2 s^{-1} (pH 3)			16
**Fe (IV)**	S77	$Fe(IV)O^{2+} + HO\bullet \rightarrow Fe(III) + H_2O$	1E7			16
**Fe (IV)**	S78	$Fe(IV)O^{2+} + H_2O_2 \rightarrow Fe(III) + HO_2 + OH^-$	1E4			16
**Fe (IV)**	S79	$Fe(IV)O^{2+} + HO_2 \rightarrow Fe(III) + O_2 + OH^-$	2E6			16
**Fe (IV)**	S80	$Fe(IV)O^{2+} + CH_3COOH \rightarrow Fe(III) + \bullet CO_2^- + prod$	3			16
**Fe (IV)**	S81	$Fe(IV)O^{2+} + H_3COOH \rightarrow Fe(III) + \bullet CO_2^- + prod$	1E2			16
**Fe (IV)**	S82	$Fe(IV)O^{2+} + Fe(II) \rightarrow Fe(III) + Fe(III)$	4E4			17

**PAA FE (II) LIGHT MECHAMISM**	S83	Fe (II) + PAA → Fe(II)-PAA	7.4E9			Assume Diffusion limited
**PAA FE (II) LIGHT MECHAMISM**	S84	Fe (II)-PAA → HO• + HO• + Fe (III) + Products	J = 8E-2 s^{-1}			This work

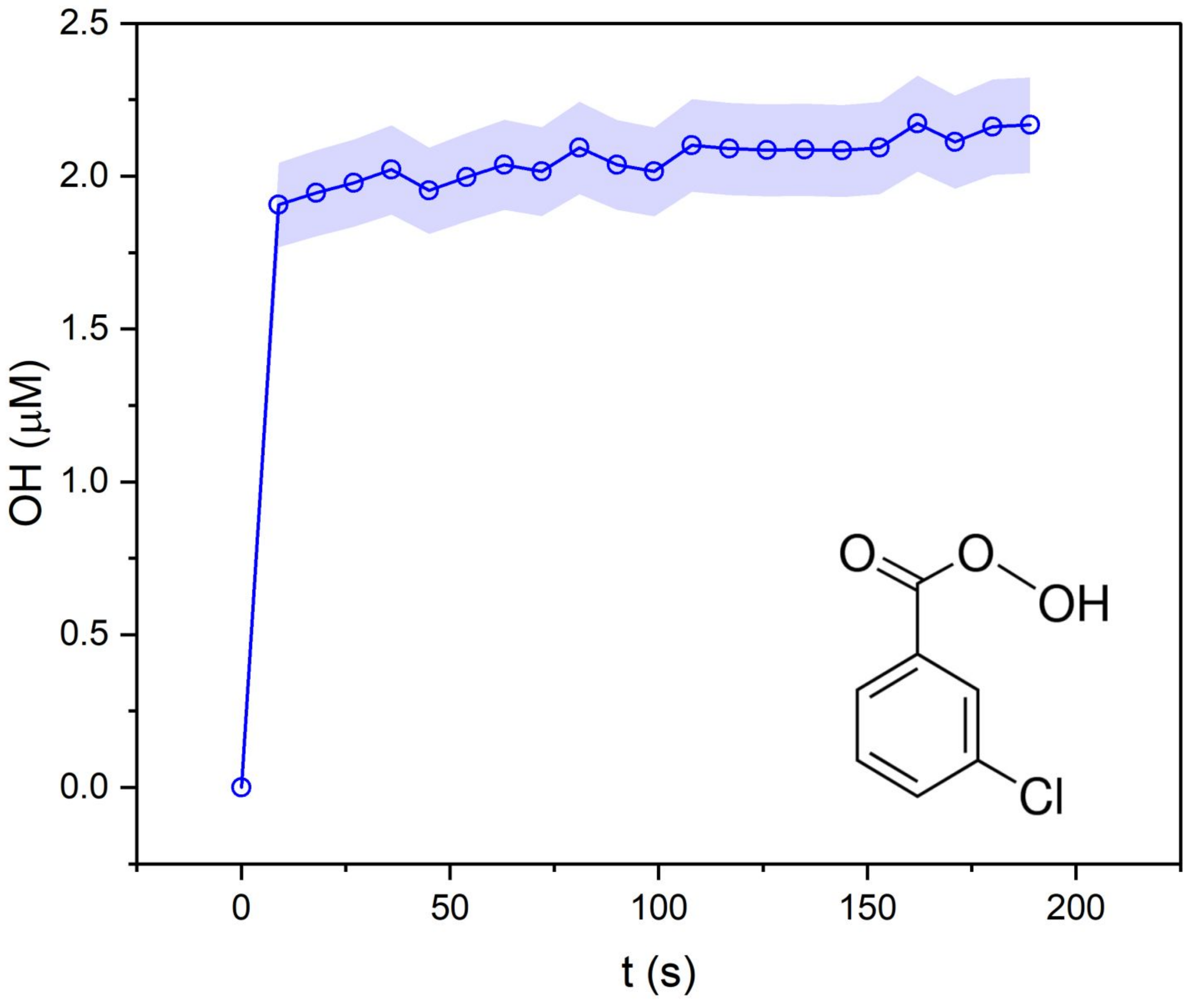

Figure S1 – OH burst observed when a 1:1 µM mixture of Fe (II) and 3-chloroperbenzoic acid is exposed to 320 nm light. Shaded area represents the standard deviation observed over three experimental repeats.

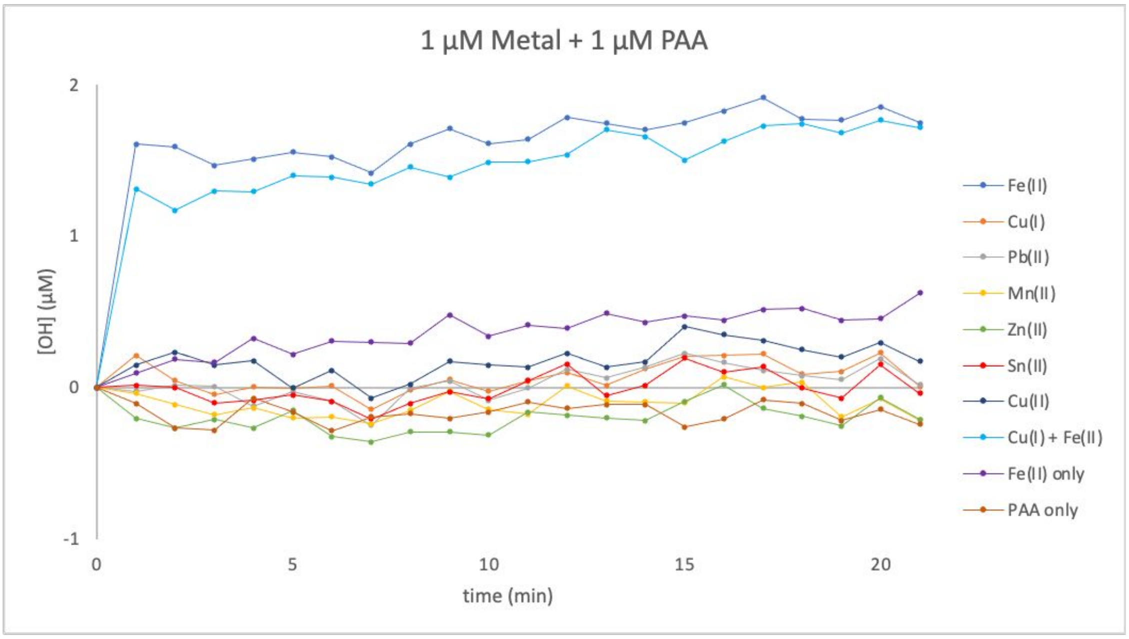

Figure S2 – Reaction of a variety of transition metals with peracetic acid. Each metal indicated was at a concentration of 1 µM.

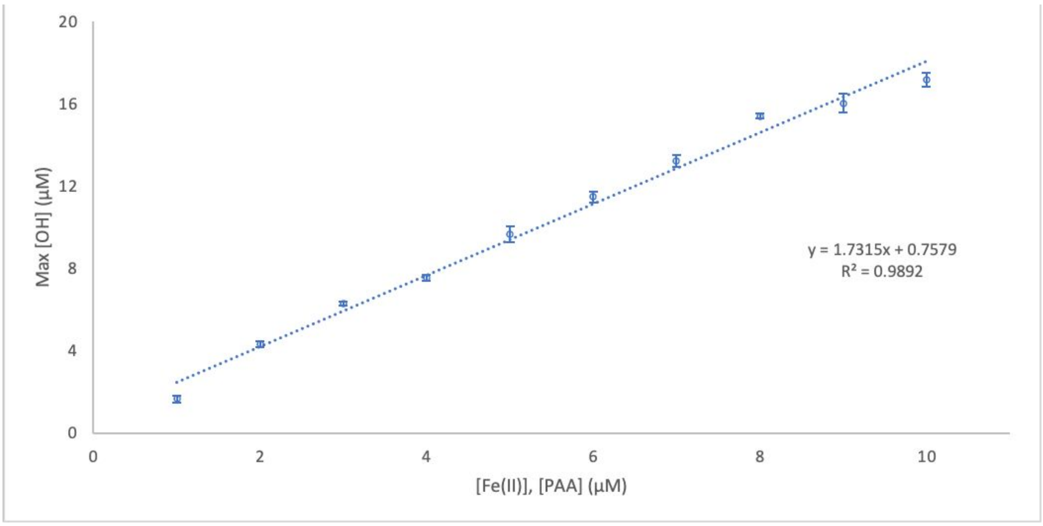

Figure S3 – Concentration dependence of light driven OH formation from Fe(II) and PAA.

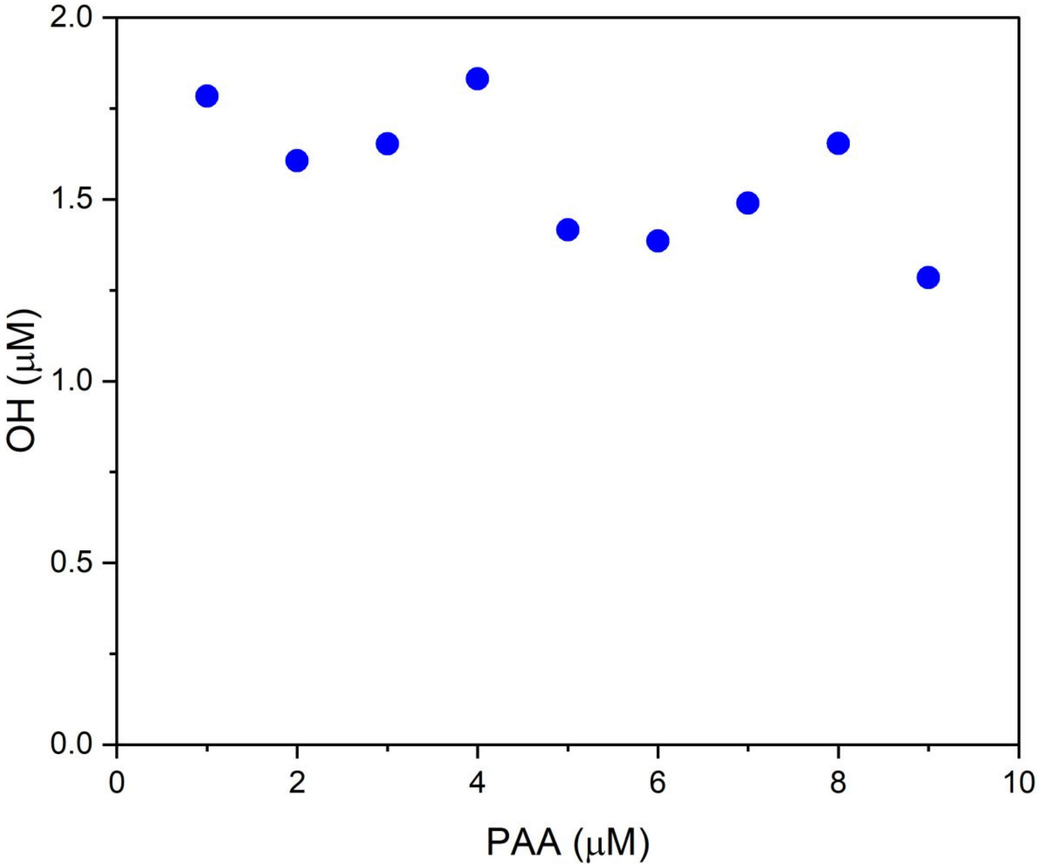

Figure S4 – Concentration dependence of the light driven Fe (II) PAA reaction, altering PAA concentrations whilst keeping Fe (II) constant at 1 μM.

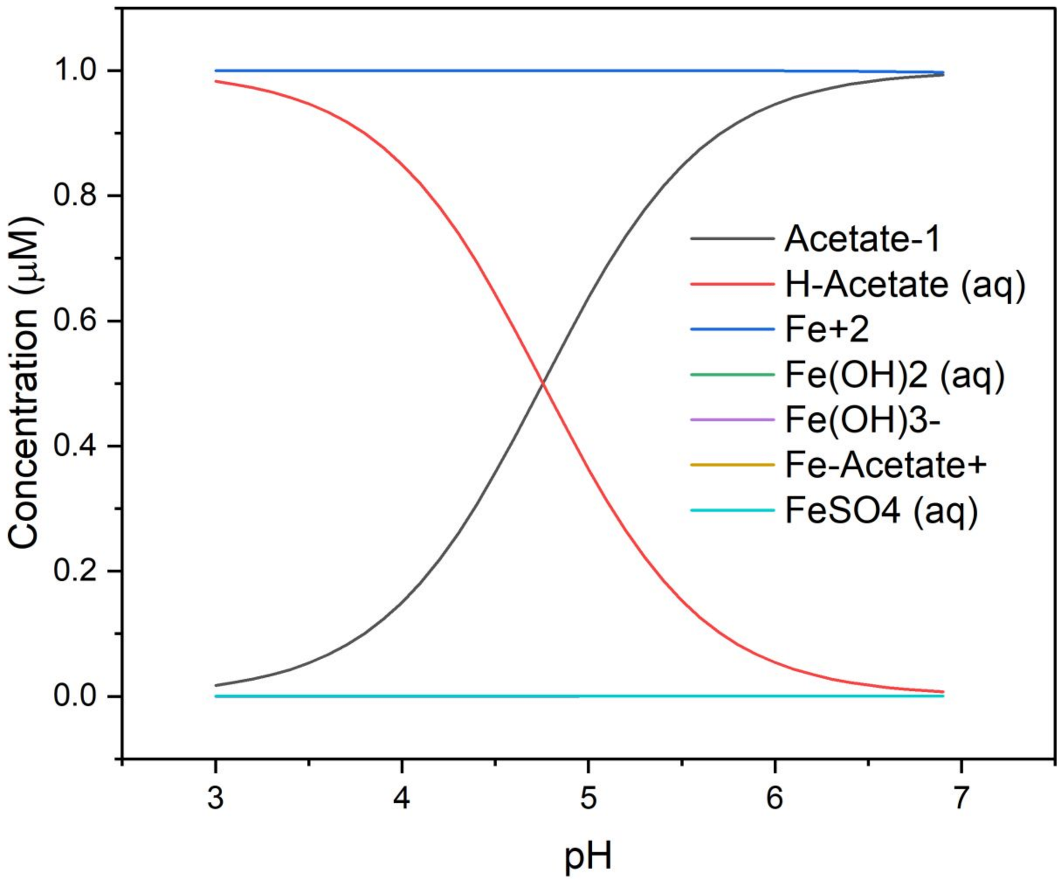

Figure S5 – MINTEQ modelling of aqueous Fe (II)

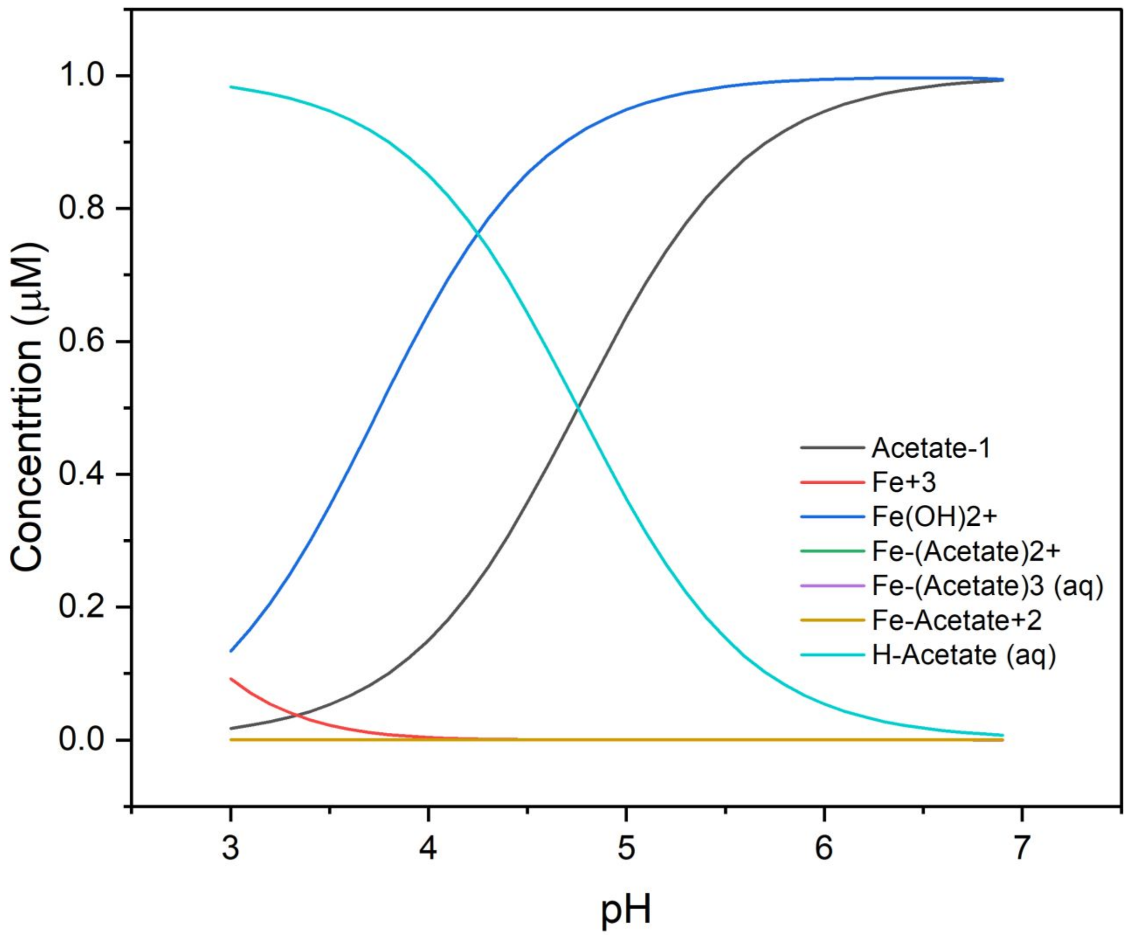

Figure S6 – MINTEQ modelling of aqueous Fe (III)

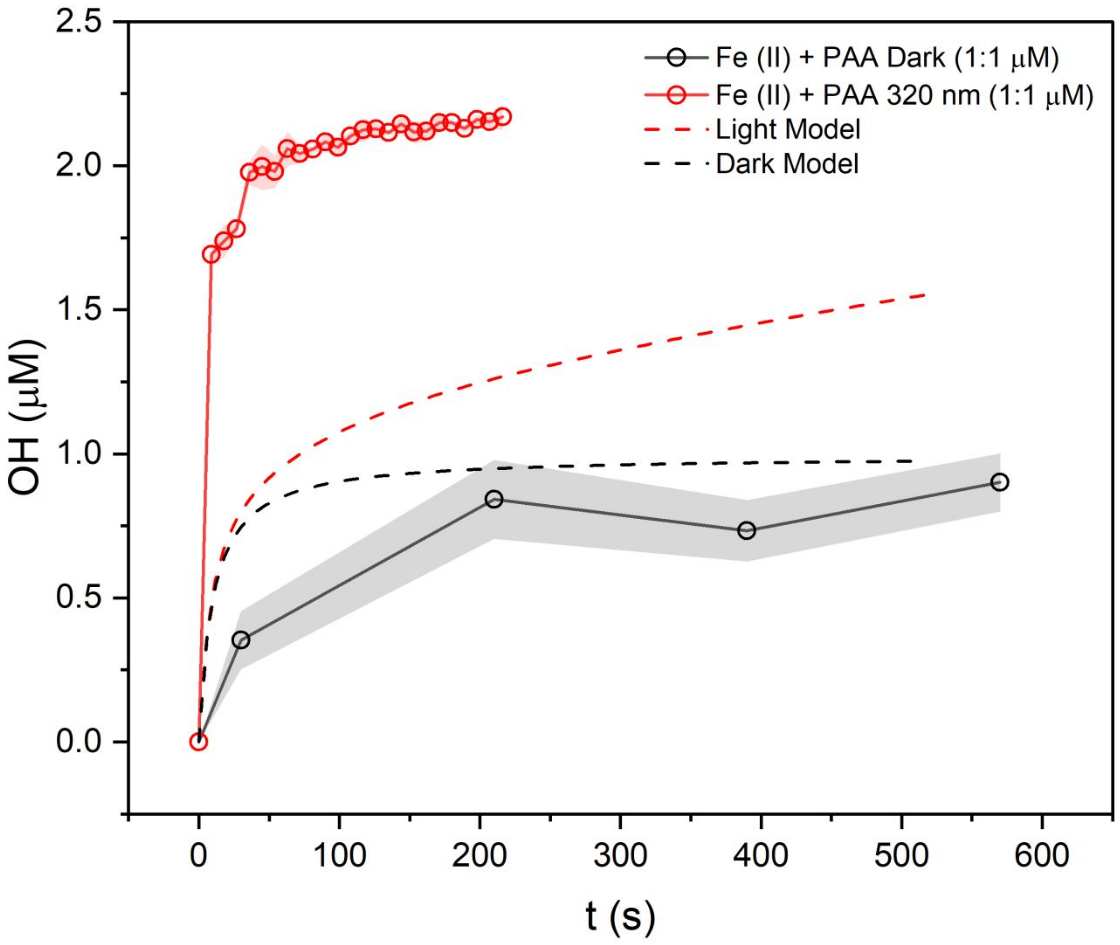

Figure S7 – Kinetic modelling showing dark reaction (black) and Fe(II)-acetate photolysis mechanism (red).

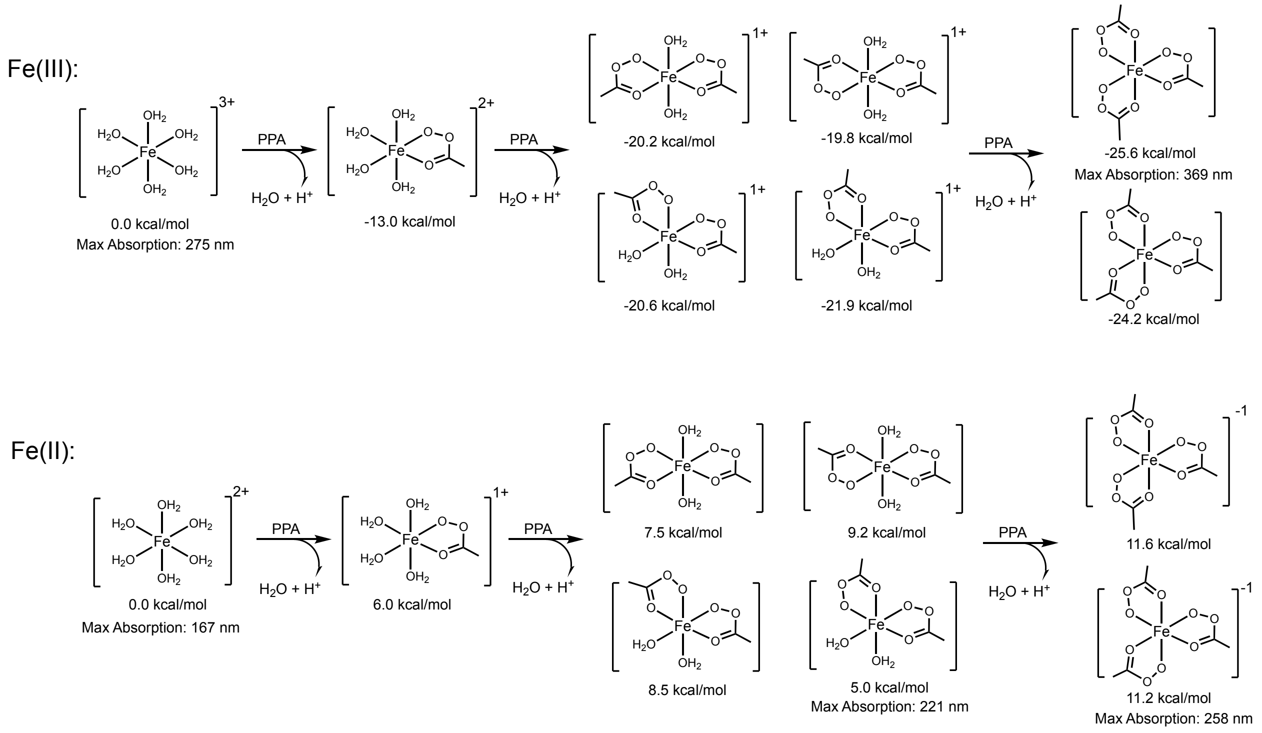

Figure S8 - Relative free energies of potential Fe(III)/Fe(II) PPA complexes. The free energies were calculated using PBE0-D3(BJ)/def2-TZVPP/SMD(water)//PBE0-D3(BJ)/def2-SVP/IEEPCM(water).

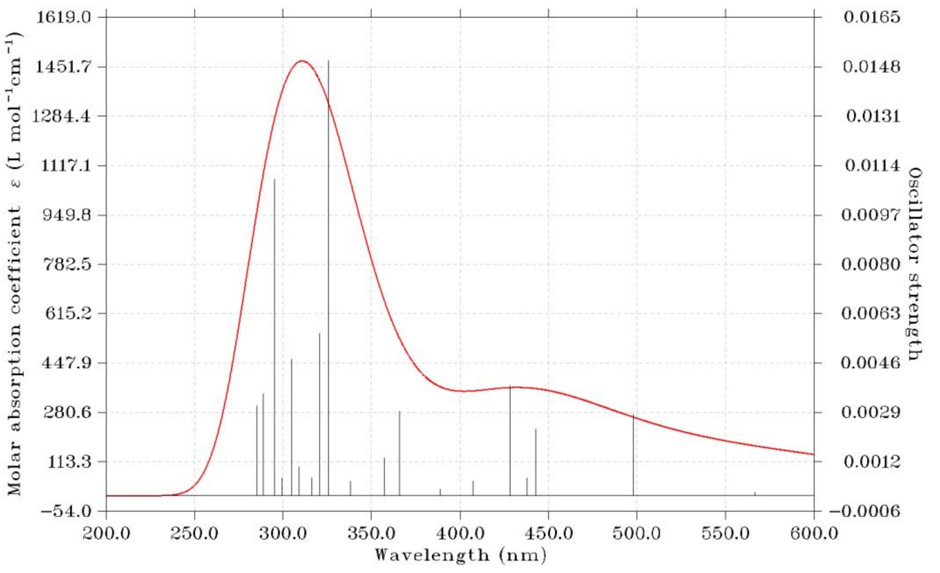

Figure S9 - Simulated UV-Vis absorption spectrum for Fe (IV) complex [Fe(IV)O(PAA)(OAc)(H2O)$_2$] using TD- PBE0-D3(BJ)/def2-TZVPP/SMD(water)

Cartesian Coordinates and Energies

For all minimum structures, no imaginary frequency was observed. Energies are reported in this section directly from the output file at the optimization level of theory (PBE0-D3(BJ)/def2-SVP/IEEPCM(water)). E_SPC, H_SPC and G_SPC are energies combining final single point energy with thermal corrections. (PBE0-D3(BJ)/def2-TZVPP/SMD(water)). All the energies here are in Hartree.

CH3COO-anion

E_SPC=-228.47917
E=-228.187889
H_SPC=-228.426119
G_SPC=-228.453467

Cartesian coordinates

C	0.199610	-0.000026	-0.000129
O	0.804239	-1.094565	0.000029
O	0.704956	1.145270	0.000023
C	-1.344688	-0.048955	-0.000051
H	-1.735743	0.480048	0.884498
H	-1.736405	0.486835	-0.880191
H	-1.730949	-1.078632	-0.003649

CH3COO-radical

E_SPC=-228.261595
E=-227.997077
H_SPC=-228.209332
G_SPC=-228.237462

Cartesian coordinates

C	0.096628	-0.003607	0.000013
O	0.820301	-1.025739	-0.000004
O	0.801874	1.035674	-0.000012
C	-1.383526	-0.010901	-0.000091
H	-1.747881	0.518859	0.891880
H	-1.748243	0.529994	-0.885121
H	-1.759885	-1.041283	-0.006159

Fe(IV)O(PAA)(OAc)(H2O)2

E_SPC=-2023.313924
E=-2022.218512
H_SPC=-2023.134415
G_SPC=-2023.194647

Cartesian coordinates

Fe	-0.041623	0.622971	-0.263624
O	0.606047	0.741752	1.671116
H	1.367884	0.046825	1.713252
O	0.661702	2.611956	-0.227122
H	0.760845	2.884673	0.696912
H	0.119397	3.283053	-0.663162
H	-0.117329	0.466527	2.250042
C	2.496072	-0.859940	0.063898
O	1.651377	-0.268533	-0.698282
O	2.452095	-0.866918	1.305265
O	-0.552162	0.657511	-1.776380
C	3.602980	-1.581881	-0.653811
H	4.137738	-0.875425	-1.305110
H	4.299153	-2.037492	0.059555
H	3.169659	-2.356302	-1.303608
C	-2.240865	-1.054573	0.102527
O	-1.032698	-1.263774	0.143159
O	-2.729668	0.162854	0.222825
O	-1.751971	1.107806	0.531328
C	-3.279738	-2.096516	-0.099373
H	-4.286223	-1.664036	-0.096058
H	-3.088352	-2.605495	-1.054540
H	-3.189036	-2.843346	0.702212

Fe(IV)OH(PAA)(OAc)(H2O)2

E_SPC=-2023.754012
E=-2022.644364
H_SPC=-2023.562534
G_SPC=-2023.62299

Cartesian coordinates
Fe -0.004851 0.557095 -0.152349
O 0.558099 0.515446 1.738756
H 1.418349 -0.072213 1.764274
O 0.738019 2.527345 -0.045950
H 0.775764 2.918393 0.839494
H 0.377147 3.198309 -0.643350
H -0.099077 0.166353 2.359375
C 2.531591 -0.858014 0.070220
O 1.564957 -0.328828 -0.638375
O 2.564793 -0.829557 1.297577
O -0.421353 0.636361 -1.848645
C 3.601318 -1.496110 -0.751462
H 4.054056 -0.735347 -1.403886
H 4.361803 -1.940552 -0.100286
H 3.153439 -2.261125 -1.401394
C -2.357713 -0.970696 0.088491
O -1.172783 -1.262461 0.157761
O -2.730219 0.310527 0.091952
O -1.684082 1.173235 0.323337
C -3.490822 -1.913982 -0.012349
H -4.433784 -1.385579 -0.188451
H -3.284529 -2.627038 -0.821556
H -3.550437 -2.477807 0.930662
H 0.307709 0.228417 -2.354515

FeII(H2O)6

E_SPC=-1721.720266
E=-1720.928857
H_SPC=-1721.551497
G_SPC=-1721.61282

Cartesian coordinates
Fe 0.002850 0.000697 -0.004199
O 1.418048 -1.178131 -1.061578
H 2.134796 -0.723447 -1.524396
O 1.336409 -0.071249 1.628972
H 1.062098 -0.032672 2.554653
O -1.343896 0.078493 -1.624550
H -2.107067 0.669741 -1.570002
O -1.414441 1.162643 1.070558
H -2.114646 0.695101 1.545395
O -1.033605 -1.727056 0.670336
H -1.697157 -2.077613 0.059626
O 1.031072 1.733172 -0.674449
H 0.580205 2.502376 -1.047625
H 1.697595 2.083229 -0.066969
H 2.099025 -0.664616 1.593314
H -1.177520 1.920168 1.621347
H -1.085742 0.048822 -2.555211
H -0.583477 -2.496081 1.045145
H 1.169096 -1.926124 -1.620408

FeII(PAA)(H2O)5

E_SPC=-1872.503846
E=-1871.564896
H_SPC=-1872.32899
G_SPC=-1872.388645

Cartesian coordinates
Fe 0.712848 0.082145 -0.002440
O 1.087414 1.877842 -1.220596
H 0.235292 2.327045 -1.316514
O 1.773726 -1.432047 1.240848
H 1.007303 -2.032597 1.217841
O 1.323284 1.329918 1.613500
H 0.664060 1.934199 1.980297
O 1.678968 -0.906314 -1.660410
H 1.766424 -0.361208 -2.454305
H 0.998865 -1.566309 -1.868380
H 2.457840 -1.887770 0.731097
H 1.671437 0.825404 2.361630
H 1.644828 2.511396 -0.747235
C -2.154824 0.051573 0.038309
O -1.269292 0.905628 0.096886
O -1.889026 -1.226160 -0.050727
O -0.515777 -1.512586 -0.094055

C	-3.610390	0.357680	0.056903
H	-4.213378	-0.555525	0.005024
H	-3.845552	0.913447	0.975569
H	-3.844268	1.010367	-0.796434

FeII(PAA)2(H2O)2-1

E_SPC=-2023.288059
E=-2022.195371
H_SPC=-2023.109108
G_SPC=-2023.168972

Cartesian coordinates

Fe	-0.053954	-0.103366	-0.001961
O	0.132840	0.612713	-2.061844
H	-0.659097	1.084910	-2.351698
O	0.117576	0.595559	2.067571
H	-0.673935	1.070999	2.353311
H	0.722237	1.278823	1.698896
H	0.729876	1.297543	-1.683767
C	2.840892	-0.332884	0.002060
O	1.856903	-1.069133	0.002894
O	2.763630	0.974400	0.006649
O	1.445028	1.482910	0.010779
C	4.244076	-0.832776	-0.006483
H	4.393932	-1.446986	-0.905918
H	4.966039	-0.008867	0.008394
H	4.393836	-1.481233	0.868337
C	-2.903799	0.009430	-0.003174
O	-2.006871	0.853586	-0.004282
O	-2.670143	-1.274592	-0.003681
O	-1.300105	-1.627433	-0.005737
C	-4.355510	0.347342	0.000558
H	-4.577492	0.970137	-0.877690
H	-4.979293	-0.553363	-0.011698
H	-4.578122	0.944804	0.896264

FeII(PAA)2(H2O)2-2

E_SPC=-2023.288359
E=-2022.196185
H_SPC=-2023.108247
G_SPC=-2023.170064

Cartesian coordinates

Fe	0.036951	0.132527	0.316299
O	-0.392061	-0.364275	2.417739
H	-0.938709	-1.083048	2.034582
O	0.049870	2.304022	0.980849
H	-0.791268	2.626293	0.628251
H	0.672120	2.384721	0.226651
H	-1.015584	0.236339	2.845797
C	2.766224	-0.399057	-0.385860
O	1.949785	-0.864218	0.406449
O	2.495168	0.605501	-1.180440
O	1.185150	1.106377	-1.046299
C	4.155163	-0.914330	-0.545318
H	4.109435	-1.974348	-0.833325
H	4.711002	-0.346275	-1.299597
H	4.669492	-0.854710	0.424373
C	-2.674160	-0.172680	-0.619144
O	-1.898413	0.761062	-0.421789
O	-2.363853	-1.414640	-0.351354
O	-1.083428	-1.581748	0.211235
C	-4.042838	-0.013408	-1.183974
H	-3.967053	0.469596	-2.168521
H	-4.553397	-0.978248	-1.278305
H	-4.620845	0.654162	-0.529046

FeII(PAA)2(H2O)2-3

E_SPC=-2023.296357
E=-2022.20378
H_SPC=-2023.115914
G_SPC=-2023.175566

Cartesian coordinates

Fe	-0.016633	-0.172642	-0.068698
O	-0.039739	0.764875	1.832383
H	0.011468	1.712310	1.502320
O	0.056139	2.901574	0.370272
H	-0.721962	2.517483	-0.089464
H	0.794805	2.468327	-0.111869
H	0.743751	0.625298	2.379592

C	2.773146	-0.739925	-0.077728
O	1.762130	-1.289362	0.359728
O	2.746182	0.398078	-0.722628
O	1.452540	0.935963	-0.888331
C	4.145135	-1.300880	0.066480
H	4.176879	-2.284466	-0.424001
H	4.898954	-0.639761	-0.375132
H	4.355874	-1.454368	1.134169
C	-2.783094	-0.749701	-0.119809
O	-1.754986	-1.394343	0.089518
O	-2.774280	0.482692	-0.559370
O	-1.479159	0.988220	-0.802264
C	-4.154693	-1.284146	0.103891
H	-4.246152	-1.593373	1.154796
H	-4.920516	-0.538079	-0.135561
H	-4.294223	-2.178318	-0.520164

FeII(PAA)2(H2O)2-4

E_SPC=-2023.288763
E=-2022.194655
H_SPC=-2023.108726
G_SPC=-2023.171602

Cartesian coordinates

Fe	0.020892	0.102252	-0.190958
O	-0.091118	-1.454650	-1.690725
H	-0.737157	-1.267214	-2.385531
O	-0.025087	1.948910	1.198254
H	-0.773910	2.178137	0.613548
H	0.754415	2.180840	0.654971
H	-0.337770	-2.323288	-1.345179
C	2.769889	-0.407780	0.356650
O	1.693857	-0.880546	0.720776
O	2.861733	0.634171	-0.427368
O	1.617794	1.193673	-0.802326
C	4.093798	-0.961001	0.758067
H	4.147048	-2.014323	0.448288
H	4.918925	-0.396156	0.310203
H	4.171880	-0.932092	1.854331
C	-2.729503	-0.376006	0.404027
O	-1.651528	-0.805558	0.812083
O	-2.827404	0.594041	-0.467384
O	-1.588418	1.142316	-0.876619
C	-4.050708	-0.906289	0.844002
H	-4.137429	-0.780636	1.932814
H	-4.878361	-0.392679	0.342348
H	-4.090327	-1.983544	0.629116

FeII(PAA)3-1

E_SPC=-2174.070596
E=-2172.809562
H_SPC=-2173.886387
G_SPC=-2173.950379

Cartesian coordinates

Fe	-0.058452	0.024877	0.429830
C	1.008401	-2.537335	-0.389802
O	0.138148	-1.788395	-0.823695
O	1.594820	-2.358627	0.765000
O	1.106120	-1.260922	1.504144
C	1.513395	-3.737172	-1.123238
H	1.914940	-3.419630	-2.096313
H	2.290234	-4.260662	-0.554035
H	0.673008	-4.418938	-1.318536
C	1.844405	2.038780	-0.447312
O	1.499812	0.932550	-0.850791
O	1.362215	2.585985	0.638025
O	0.436579	1.787857	1.338211
C	2.850167	2.900713	-1.138591
H	2.515604	3.087809	-2.169006
H	2.992764	3.853188	-0.615153
H	3.806847	2.361192	-1.193884
C	-2.766887	0.417187	-0.495822
O	-1.654396	0.679654	-0.943706
O	-2.961753	-0.087679	0.694014
O	-1.786715	-0.272002	1.457665
C	-4.036773	0.635673	-1.252550
H	-4.912261	0.325116	-0.670773
H	-4.121416	1.701214	-1.510863
H	-3.994858	0.069448	-2.194069

FeII(PAA)3

E_SPC=-2174.070346
E=-2172.809912
H_SPC=-2173.885116
G_SPC=-2173.95115

Cartesian coordinates

Fe	-0.157772	-0.067093	0.141866
C	1.430658	2.365275	-0.253194
O	1.150224	1.394542	-0.945376
O	0.940048	2.551580	0.946659
O	0.062954	1.540806	1.380029
C	2.360340	3.456074	-0.676944
H	1.982631	3.910002	-1.604379
H	2.459982	4.224905	0.098054
H	3.345960	3.021798	-0.898338
C	-2.999593	-0.088523	-0.257804
O	-2.050602	0.408020	-0.860054
O	-2.882355	-0.774057	0.846838
O	-1.561185	-0.901807	1.343220
C	-4.416383	0.030023	-0.719785
H	-4.497937	-0.385422	-1.734456
H	-5.106259	-0.492560	-0.047284
H	-4.686434	1.094517	-0.775477
C	1.983727	-2.019386	0.062847
O	1.589721	-1.192254	0.878281
O	1.366841	-2.263783	-1.064707
O	0.210952	-1.493124	-1.284193
C	3.211705	-2.850599	0.247020
H	3.367721	-3.532088	-0.597204
H	4.081466	-2.186215	0.353512
H	3.119444	-3.427110	1.178634

FeIII(H2O)6

E_SPC=-1721.518868
E=-1720.682852
H_SPC=-1721.350615
G_SPC=-1721.408217

Cartesian coordinates

Fe	0.000029	0.000073	-0.000112
O	-1.398317	0.631890	-1.291374
H	-2.239448	1.048483	-1.041424
O	0.879348	1.799668	-0.059126
H	1.671250	2.056037	0.441648
O	-0.879043	-1.799413	0.059776
H	-0.577925	-2.559064	0.584569
O	1.398045	-0.632226	1.290656
H	2.239405	-1.048278	1.040561
O	1.154351	-0.607919	-1.519900
H	1.108929	-1.477666	-1.950186
O	-1.154307	0.607728	1.520516
H	-1.809497	0.061193	1.984894
H	-1.109051	1.477398	1.951001
H	0.578429	2.559896	-0.583244
H	1.344308	-0.590354	2.259664
H	-1.670119	-2.056826	-0.441831
H	1.807029	-0.060465	-1.986765
H	-1.344687	0.589922	-2.260356

FeIII(OAc)(PAA)(H2O)2

E_SPC=-1948.047305
E=-1947.022522
H_SPC=-1947.870492
G_SPC=-1947.932293

Cartesian coordinates

Fe	-0.215809	-0.551333	-0.021654
O	-0.129464	-1.677777	1.714156
H	-0.663548	-1.345205	2.451404
O	-1.329384	-2.119428	-0.751858
H	-1.224531	-3.017543	-0.405304
H	-1.605663	-2.185640	-1.677337
H	0.702828	-2.006945	2.083791
C	-2.018852	1.086094	-0.056260
O	-1.687484	0.545399	1.034373
O	-1.368502	0.731212	-1.097599
C	-3.101476	2.098149	-0.151003
H	-2.662800	3.055648	-0.469272
H	-3.818631	1.793445	-0.926024
H	-3.605878	2.221463	0.813324
C	2.268176	0.748891	0.034618

O 1.173394 0.866345 0.589551
O 2.472025 -0.221499 -0.827402
O 1.356178 -1.023352 -1.001881
C 3.431689 1.635174 0.250156
H 4.291698 1.313519 -0.346570
H 3.142479 2.660721 -0.020770
H 3.683762 1.628136 1.319992

FeIII(PAA)(H2O)5

E_SPC=-1872.330597
E=-1871.361392
H_SPC=-1872.155318
G_SPC=-1872.214259

Cartesian coordinates
Fe 0.739941 0.052112 -0.023345
O 1.131809 2.104075 -0.153808
H 0.375861 2.704381 -0.062991
O 2.080310 -1.514396 0.214574
H 1.745164 -2.409599 0.049420
O 1.063570 0.290644 1.996394
H 0.500065 0.841428 2.559771
O 0.991649 0.155438 -2.038671
H 1.208165 0.985633 -2.487586
H 0.613253 -0.460492 -2.682300
H 2.978230 -1.468882 -0.148116
H 1.333573 -0.481300 2.515804
H 1.877468 2.504629 0.317816
C -2.074599 0.014510 0.039294
O -1.163931 0.846620 0.089582
O -1.776009 -1.260939 -0.063319
O -0.416586 -1.472338 -0.098424
C -3.517421 0.317436 0.079169
H -4.113727 -0.600592 0.054877
H -3.729112 0.891251 0.992375
H -3.761791 0.954123 -0.783514

FeIII(PAA)2(H2O)2-1

E_SPC=-2023.132362
E=-2022.021036
H_SPC=-2022.951191
G_SPC=-2023.01173

Cartesian coordinates
Fe 0.002589 0.394411 0.040731
O -0.165754 0.928265 2.046327
H 0.410218 0.452487 2.662447
O -0.381918 2.386655 -0.485574
H -1.296097 2.614668 -0.708745
H 0.188736 2.737414 -1.184904
H -1.049143 0.952664 2.441359
C 2.671875 -0.499384 0.026431
O 1.736021 -0.435876 0.829947
O 2.546724 -0.000466 -1.175391
O 1.310194 0.595336 -1.389974
C 3.981755 -1.127451 0.309347
H 3.817105 -2.191621 0.531929
H 4.665961 -1.021083 -0.538984
H 4.409077 -0.659371 1.207158
C -2.667441 -0.573918 -0.132337
O -2.123238 0.509512 0.091552
O -1.949232 -1.659123 -0.279637
O -0.584367 -1.432492 -0.155358
C -4.127248 -0.776624 -0.271364
H -4.483436 -0.161248 -1.110160
H -4.367746 -1.830229 -0.448301
H -4.623076 -0.418594 0.641598

FeIII(PAA)2(H2O)2-2

E_SPC=-2023.133222
E=-2022.022277
H_SPC=-2022.952247
G_SPC=-2023.013873

Cartesian coordinates
Fe -0.010455 0.557934 0.001191
O -0.146112 1.443485 1.887632
H 0.480929 1.135314 2.558364
O 0.786969 2.392272 -0.514846
H 0.483289 3.206984 -0.089673
H 0.994851 2.600009 -1.436801

H	-0.995728	1.573720	2.332290
C	2.405484	-0.862587	0.077933
O	1.651280	-0.322296	0.891057
O	2.100955	-0.890512	-1.196396
O	0.878557	-0.289946	-1.480945
C	3.688233	-1.516714	0.418649
H	3.485761	-2.331737	1.128284
H	4.185489	-1.908108	-0.475043
H	4.331963	-0.783457	0.924618
C	-2.387311	-0.911899	0.077877
O	-1.267592	-0.978397	0.589614
O	-2.702943	0.091703	-0.706505
O	-1.672210	1.012346	-0.848180
C	-3.467195	-1.905341	0.269255
H	-3.697806	-1.968790	1.342264
H	-4.364402	-1.630785	-0.295380
H	-3.099018	-2.889426	-0.053619

FeIII(PAA)2(H2O)2-3

E_SPC=-2023.130891
E=-2022.015529
H_SPC=-2022.949819
G_SPC=-2023.011198

Cartesian coordinates

Fe	0.002585	-0.544491	-0.025299
O	-0.101416	-0.621250	2.070900
H	-0.786505	-1.216462	2.408748
O	0.069501	-0.364074	-2.115820
H	-0.731109	-0.461347	-2.650647
H	0.752891	-0.902629	-2.541267
H	0.684894	-0.761079	2.617217
C	2.527547	0.710393	0.080183
O	1.345370	1.041753	0.183491
O	2.854348	-0.547368	-0.095411
O	1.752597	-1.392049	-0.145529
C	3.678046	1.640270	0.131145
H	3.616559	2.226782	1.058159
H	4.628872	1.098955	0.082785
H	3.598649	2.338368	-0.714846
C	-2.520001	0.718599	-0.006312
O	-1.336730	1.057177	-0.063312
O	-2.849459	-0.549715	0.042096
O	-1.749229	-1.399499	0.012601
C	-3.668576	1.652427	0.006410
H	-3.647430	2.242345	-0.921227
H	-4.618791	1.114392	0.089238
H	-3.547198	2.347501	0.848917

FeIII(PAA)2(H2O)2-4

E_SPC=-2023.13103
E=-2022.016373
H_SPC=-2022.949952
G_SPC=-2023.010483

Cartesian coordinates

Fe	-0.017167	0.301958	0.031022
O	-0.141070	1.090964	-1.881040
H	-0.971348	1.290261	-2.336200
O	-0.030395	0.395556	2.104853
H	-0.849587	0.449955	2.617503
H	0.583501	1.048664	2.472266
H	0.465351	1.829527	-2.039747
C	2.695787	-0.499764	-0.068173
O	1.579956	-1.020500	-0.141818
O	2.814514	0.794247	0.087441
O	1.587452	1.439165	0.154010
C	3.977291	-1.237311	-0.138092
H	4.012086	-1.793306	-1.085536
H	4.832176	-0.556675	-0.065229
H	4.004304	-1.969209	0.681838
C	-2.792520	-0.224299	-0.000227
O	-2.052078	0.744860	0.193013
O	-2.285821	-1.401371	-0.259468
O	-0.894899	-1.407250	-0.277124
C	-4.271182	-0.174773	0.034334
H	-4.614598	0.552230	-0.715390
H	-4.706543	-1.159633	-0.164687
H	-4.586534	0.188799	1.022639

FeIII(PAA)3-1

E_SPC=-2173.928229
E=-2172.670061
H_SPC=-2173.741648
G_SPC=-2173.805133

Cartesian coordinates

Fe	-0.004122	0.005072	0.314707
C	0.445620	-2.678943	-0.403268
O	-0.044407	-1.684050	-0.937369
O	1.006535	-2.609613	0.778675
O	1.012278	-1.320168	1.314196
C	0.460711	-4.036110	-1.002441
H	0.992594	-3.990567	-1.963374
H	0.944970	-4.759762	-0.337987
H	-0.574756	-4.342816	-1.206956
C	2.107526	1.719481	-0.407489
O	1.473449	0.811149	-0.944525
O	1.784193	2.163187	0.781701
O	0.657796	1.535911	1.318494
C	3.278385	2.400654	-1.012533
H	2.969657	2.846906	-1.968538
H	3.677846	3.173892	-0.347555
H	4.051154	1.649189	-1.227651
C	-2.553826	0.954709	-0.403116
O	-1.445563	0.886501	-0.934830
O	-2.773336	0.438238	0.780747
O	-1.656348	-0.203118	1.320231
C	-3.739603	1.609705	-1.007803
H	-4.610551	1.546474	-0.346695
H	-3.495387	2.661388	-1.213732
H	-3.957997	1.122151	-1.968518

FeIII(PAA)3

E_SPC=-2173.926592
E=-2172.665845
H_SPC=-2173.740999
G_SPC=-2173.802956

Cartesian coordinates

Fe	-0.067477	-0.051899	-0.049975
C	0.964564	2.582749	0.113460
O	0.570959	1.728038	0.905973
O	1.033314	2.335782	-1.171376
O	0.674451	1.028268	-1.497617
C	1.392362	3.952282	0.492577
H	2.245612	3.875692	1.181581
H	1.673713	4.539776	-0.388061
H	0.570034	4.444252	1.030511
C	2.034881	-1.902214	0.193475
O	1.645330	-0.899586	0.795594
O	1.341579	-2.412908	-0.790096
O	0.142303	-1.738881	-1.027293
C	3.295161	-2.624348	0.494271
H	3.269379	-2.956746	1.541655
H	3.429620	-3.484138	-0.170800
H	4.136422	-1.925465	0.384798
C	-2.860793	-0.372547	-0.007576
O	-1.998784	-0.072917	-0.838093
O	-2.554412	-0.569063	1.246401
O	-1.199088	-0.399994	1.523689
C	-4.301480	-0.537903	-0.319794
H	-4.875708	-0.796694	0.576257
H	-4.678282	0.399491	-0.752945
H	-4.409764	-1.324831	-1.079569

H2O

E_SPC=-76.392968
E=-76.283367
H_SPC=-76.367679
G_SPC=-76.386097

Cartesian coordinates

O	0.000000	0.000000	0.120595
H	-0.000000	0.752254	-0.482382
H	-0.000000	-0.752254	-0.482382

OH-anion

E_SPC=-75.885833
E=-75.715678
H_SPC=-75.874186
G_SPC=-75.890739

Cartesian coordinates

O	0.000000	0.000000	0.108042
H	0.000000	0.000000	-0.864337

OH-radical

E_SPC=-75.695534
E=-75.592388
H_SPC=-75.683696
G_SPC=-75.700913

Cartesian coordinates

O	0.000000	0.000000	0.108600
H	-0.000000	-0.000000	-0.868797

PAA

E_SPC=-304.028355
E=-303.665744
H_SPC=-303.956452
G_SPC=-303.988299

Cartesian coordinates

C	-0.411972	0.163746	-0.000344
O	-0.100419	1.332377	-0.000076
O	0.527953	-0.795232	-0.000537
O	1.820745	-0.244305	0.000486
C	-1.785129	-0.409055	0.000082
H	-2.324298	-0.032155	-0.880309
H	-1.767256	-1.504652	-0.012544
H	-2.314110	-0.053728	0.895688
H	1.602045	0.719672	-0.000244

REFERENCES

(1) Kim, J.; Zhang, T.; Liu, W.; Du, P.; Dobson, J. T.; Huang, C. H. Advanced Oxidation Process with Peracetic Acid and Fe(II) for Contaminant Degradation. *Environ. Sci. Technol.* **2019**, *53* (22), 13312–13322.

(2) Cai, M.; Sun, P.; Zhang, L.; Huang, C. H. UV/Peracetic Acid for Degradation of Pharmaceuticals and Reactive Species Evaluation. *Environ. Sci. Technol.* **2017**, *51* (24), 14217–14224.

(3) Kamath, D.; Mezyk, S. P.; Minakata, D. Elucidating the Elementary Reaction Pathways and Kinetics of Hydroxyl Radical-Induced Acetone Degradation in Aqueous Phase Advanced Oxidation Processes. *Environ. Sci. Technol.* **2018**, *52* (14), 7763–7774.

(4) Schuchmann, H. P.; Clemens von, S. A Study of the Y-Radiolysis of Methane in Oxygenated Aqueous Solutions. *Zeitschrift fur Naturforsch. - Sect. B J. Chem. Sci.* **1984**, *39* (9), 1262–1267.

(5) Neta, P.; Huie, R. E.; Ross, A. B. Rate Constants for Reactions of Peroxyl Radicals in Fluid Solutions. *J. Phys. Chem. Ref. Data* **1990**, *19* (2), 413–513.

(6) De Laat, J.; Gallard, H. Catalytic Decomposition of Hydrogen Peroxide by Fe(III) in Homogeneous Aqueous Solution: Mechanism and Kinetic Modeling. *Environ. Sci. Technol.* **1999**, *33* (16), 2726–2732.

(7) He, J.; Yang, X.; Men, B.; Wang, D. Interfacial Mechanisms of Heterogeneous Fenton Reactions Catalyzed by Iron-Based Materials: A Review. *J. Environ. Sci. (China)* **2016**, *39*, 97–109.

(8) Pignatello, J. J.; Oliveros, E.; MacKay, A. Advanced Oxidation Processes for Organic Contaminant Destruction Based on the Fenton Reaction and Related Chemistry. *Crit. Rev. Environ. Sci. Technol.* **2006**, *36* (1), 1–84.

(9) De Laat, J.; Gallard, H.; Ancelin, S.; Legube, B. Comparative Study of the Oxidation of Atrazine and Acetone by H2O2/UV, Fe(III)/UV, Fe(III)/H2O2/UV and Fe(II) or Fe(III)/H2O2. *Chemosphere* **1999**, *39* (15), 2693–2706.

(10) Rokhina, E. V.; Makarova, K.; Lahtinen, M.; Golovina, E. A.; Van As, H.; Virkutyte, J. Ultrasound-Assisted MnO2 Catalyzed Homolysis of Peracetic Acid for Phenol Degradation: The Assessment of Process Chemistry and Kinetics. *Chem. Eng. J.* **2013**, *221*, 476–486.

(11) Rojas, M. R.; Pérez, F.; Whitley, D.; Arnold, R. G.; Sáez, A. E. Modeling of Advanced Oxidation of Trace Organic Contaminants by Hydrogen Peroxide Photolysis and Fentons Reaction. *Ind. Eng. Chem. Res.* **2010**, *49* (22), 11331–11343.

(12) Mártire, D. O.; Caregnato, P.; Furlong, J.; Allegretti, P.; Gonzalez, M. C. Kinetic Study of the Reactions of Oxoiron(IV) with Aromatic Substrates in Aqueous Solutions. *Int. J. Chem. Kinet.* **2002**, *34* (8), 488–494.

(13) Gonzalez, D. H.; Cala, C. K.; Peng, Q.; Paulson, S. E. HULIS Enhancement of Hydroxyl Radical Formation from Fe(II): Kinetics of Fulvic Acid-Fe(II) Complexes in the Presence of Lung Antioxidants. *Environ. Sci. Technol.* **2017**, *51* (13), 7676–7685.

(14) De Laat, J.; Le, T. G. Kinetics and Modeling of the Fe(III)/H2O2 System in the Presence of Sulfate in Acidic Aqueous Solutions. *Environ. Sci. Technol.* **2005**, *39* (6), 1811–1818.

(15) Pandey, R. N.; Smith, W. M. Carboxylate Complexing of Iron(III). I. The Formation of Monoacetatoiron(III) in Aqueous Solution: Equilibria and Kinetics. *Can. J. Chem.* **1972**, *50* (2), 194–200.

(16) Jacobsen, F.; Holcman, J.; Sehested, K. Reactions of the Ferryl Ion with Some Compounds Found in Cloud Water. *Int. J. Chem. Kinet.* **1998**, *30* (3), 215–221.

(17) Pestovsky, O.; Bakac, A. Reactivity of Aqueous Fe(IV) in Hydride and Hydrogen Atom Transfer Reactions. *J. Am. Chem. Soc.* **2004**, *126* (42), 13757–13764.

(18) Page, S. E.; Arnold, W. A.; McNeill, K. Terephthalate as a Probe for Photochemically Generated Hydroxyl Radical. *J. Environ. Monit.* **2010**, *12* (9), 1658–1665.

(19) Sedlak, D. L.; Hoigné, J. The Role of Copper and Oxalate in the Redox Cycling of Iron in Atmospheric Waters. *Atmos. Environ. Part A, Gen. Top.* **1993**, *27* (14), 2173–2185.

(20) Rohatgi, N. K. Cross Sections of H202 Vapor (X 1020cm 2 Fe. **1978**, *5* (2), 113–115.

(21) Zhang, T.; Huang, C. H. Modeling the Kinetics of UV/Peracetic Acid Advanced Oxidation Process. *Environ. Sci. Technol.* **2020**, *54* (12), 7579–7590.

(22) Benkelberg, H. J.; Warneck, P. Photodecomposition of Iron(III) Hydroxo and Sulfato Complexes in Aqueous Solution: Wavelength Dependence of OH and 804- Quantum Yields. *J. Phys. Chem.* **1995**, *99* (14), 5214–5221.
